# A hybrid-hierarchical genome assembly strategy to sequence the invasive golden mussel, *Limnoperna fortunei*

**DOI:** 10.1093/gigascience/gix128

**Published:** 2017-12-15

**Authors:** Marcela Uliano-Silva, Francesco Dondero, Thomas Dan Otto, Igor Costa, Nicholas Costa Barroso Lima, Juliana Alves Americo, Camila Junqueira Mazzoni, Francisco Prosdocimi, Mauro de Freitas Rebelo

**Affiliations:** 1 Carlos Chagas Filho Biophysics Institute (IBCCF), Universidade Federal do Rio de Janeiro, Rio de Janeiro, Brazil; 2 Department of Evolutionary Genetics, Leibniz Institute for Zoo and Wildlife Research, Berlin, Germany; 3 Berlin Center for Genomics in Biodiversity Research, Berlin, Germany; 4 Department of Science and Technological Innovation (DiSIT), Università del Piemonte Orientale Amedeo Avogadro, Vercelli-Novara-Alessandria, Italy; 5 Wellcome Trust Sanger Institute, Wellcome Genome Campus, Hinxton CB10 1SA, UK; 6 Centre of Immunobiology, Institute of Infection, Immunity & Inflammation, College of Medical, Veterinary and Life Sciences, University of Glasgow, Glasgow, UK; 7 Leopoldo de Meis Biomedical Biochemistry Institute (IBqM), Universidade Federal do Rio de Janeiro, Rio de Janeiro, Brazil; 8 Bioinformatics Laboratory (LabInfo) of the National Laboratory for Scientific Computing, Petrópolis, Rio de Janeiro, Brazil

**Keywords:** Amazon, binding domain, bivalves, genomics, TLR, transposon

## Abstract

**Background:**

For more than 25 years, the golden mussel, *Limnoperna fortunei*, has aggressively invaded South American freshwaters, having travelled more than 5000 km upstream across 5 countries. Along the way, the golden mussel has outcompeted native species and economically harmed aquaculture, hydroelectric powers, and ship transit. We have sequenced the complete genome of the golden mussel to understand the molecular basis of its invasiveness and search for ways to control it.

**Findings:**

We assembled the 1.6-Gb genome into 20 548 scaffolds with an N50 length of 312 Kb using a hybrid and hierarchical assembly strategy from short and long DNA reads and transcriptomes. A total of 60 717 coding genes were inferred from a customized transcriptome-trained AUGUSTUS run. We also compared predicted protein sets with those of complete molluscan genomes, revealing an exacerbation of protein-binding domains in *L. fortunei*.

**Conclusions:**

We built one of the best bivalve genome assemblies available using a cost-effective approach using Illumina paired-end, mate-paired, and PacBio long reads. We expect that the continuous and careful annotation of *L. fortunei*’s genome will contribute to the investigation of bivalve genetics, evolution, and invasiveness, as well as to the development of biotechnological tools for aquatic pest control.

## Data Description

The golden mussel *Limnoperna fortunei* is an Asian bivalve that arrived in the southern part of South America about 25 years ago [1]. Research suggests that *L. fortunei* was introduced in South America through ballast water of ships coming from Hong Kong or Korea [2]. It was found for the first time in the estuary of the La Plata River in 1991 [1]. Since then, it has moved ∼5000 km, invading upstream continental waters and reaching northern parts of the continent [3], leaving behind a track of great economic impact and environmental degradation [4]. The latest infestation was reported in 2016 in the São Francisco River, one of the main rivers in the northeast of Brazil, with a 2700-km riverbed that provides water to more than 14 million people. At Paulo Afonso, one of the main hydroelectric power plants in the São Francisco River, maintenance due to clogging of pipelines and corrosion caused by the golden mussel is estimated to cost U$700 000 per year (personal communication, Mizael Gusmã, Chief Maintenance Engineer for Centrais Hidrelétricas do São Francisco [CHESF]).

A recent review has shown that, before arriving in South America, *L. fortunei* was already an invader in China. Originally from the Pearl River Basin, the golden mussel has traveled 1500 km into the Yang Tse and Yellow River basins, being limited further north only by the extreme natural barriers of Northern China [5]. Today, *L. fortunei* is found in the Paraguaizinho River, located only 150 km from the Teles-Pires River that belongs to the Alto Tapajós River Basin and is the first to directly connect with the Amazon River Basin [6]. Due to its fast dispersion rates, it is very likely that *L. fortunei* will reach the Amazon River Basin in the near future.

The reason why some freshwater bivalves, such as *L. fortunei, Dreissena polymorpha*, and *Corbicula fluminea*, are aggressive invaders is not fully understood. These bivalves present characteristics such as (i) tolerance to a wide range of environmental variables, (ii) short life span, (iii) early sexual maturation, and (iv) high reproductive rates that allow them to reach densities as high as 150 000 ind.m^−2^ over a year [7, 8] that may explain the aggressive behavior. On the other hand, these traits are not exclusive to invasive freshwater bivalves and do not explain how they outcompete native species and disperse so widely.

To the best of our knowledge, there are no reports of strategies successful at controlling the expansion of mussel invasion in industrial facilities. Bivalves can sense chemicals in the water and close their valves as a defensive response [9], making them tolerant to a wide range of chemical substances, including strong oxidants like chlorine [10]. Microencapsulated chemicals have shown better results in controlling mussel populations in closed environments [10, 11], but it is unlikely they would work in the wild. Currently, there is no effective and efficient approach to control the invasion by *L. fortunei*.

The genome sequence is one of the most relevant and informative descriptions of species biology. The genetic substrate of invasive populations, upon which natural selection operates, can be of primary importance to understanding and controlling a biological invader [12, 13].

We have partially funded the golden mussel genome sequencing through a pioneer crowdfunding initiative in Brazil [14]. In this campaign, we were able to raise around USD$20 000.00 at the same time as we promoted scientific education and awareness in Brazil.

Here we present the first complete genome dataset for the invasive bivalve *Limnoperna fortunei*, assembled from short and long DNA reads and using a hybrid and hierarchical assembly strategy. This high-quality reference genome represents a substantial resource for further studies of genetics and evolution of mussels, as well as for the development of new tools for plague control.

### Genome sequencing in short Illumina and long PacBio reads

*Limnoperna fortunei* mussels were collected from the Jacui River, Porto Alegre, Rio Grande do Sul, Brazil (29°59΄29.3″S 51°16΄24.0″W). Voucher specimens were housed at the zoological collection (specimen number: 19 643) of the Biology Institute at the Universidade Federal do Rio de Janeiro, Brazil. For the genome assembly, a total of 3 individuals were sampled for DNA extraction from gills and to produce the 3 types of DNA libraries used in this study. DNA was extracted using DNeasy Blood and Tissue Kit (Qiagen, Hilden, Germany) to prepare libraries for Illumina Nextera paired-end reads, with ∼180-bp and ∼500-bp insert sizes, (ii) Illumina Nextera mate-paired reads with insert sizes ranging from 3 to 15 Kb, and (iii) Pacific Biosciences long reads (Table 1). Illumina libraries were sequenced, respectively, in a HiScanSQ or HiSeq 1500 machine, and Pacific Biosciences reads were produced with the P4C6 chemistry and sequenced in 10-SMRT Cells. All Illumina reads were submitted to quality analysis with FastQC (FastQC, RRID:SCR_014583) followed by trimming with Trimmomatic (Trimmomatic, RRID:SCR_011848) [15]. Pacific Biosciences adaptor-free subread sequences were used as input data for the genome assembly.

**Table 1:** DNA reads produced for *L. fortunei* genome assembly

Library technology			Raw data		Trimmed data*	
	Reads insert size	Pairs	Number of reads	Number of bases	Number of reads	Number of bases
Illumina Nextera	Paired-end – 180 bp	R1	209 542 721	21 060 365 702	209 036 571	21 001 101 404
		R2	209 542 721	21 049 308 698	209 036 571	20 991 650 008
	Paired-end – 500 bp	R1	153 948 902	15 472 966 961	153 482 290	15 423 123 500
		R2	153 948 902	15 462 883 157	153 482 290	15 414 813 589
	Mate-paired 3 – 12 Kb	R1	178 392 944	18 017 687 344	58 157 933	5 822 572 152
		R2	178 392 944	18 017 687 344	58 157 933	5 811 310 412
Pacific Biosciences	P4C – 10/SMTRC	Subreads	1 663 730	11 171 487 485		

*Trimmomatic parameters for Illumina reads—ILLUMINACLIP: NexteraPE-PE.fa:2:30:10 SLIDINGWINDOW:4:2 LEADING:10 TRAILING:10 CROP:101 HEADCROP:0 MINLEN:80.

For transcriptome sequencing, RNA was sampled from 4 tissues (gills, adductor muscle, digestive gland, and foot) of 3 different golden mussel specimens. RNA was extracted using the NEXTflex Rapid Directional RNA-Seq Kit (Bio Scientifics, TX, USA) and 12 barcodes from NEXTflex Barcodes compatible with Illumina NexSeq Machine. Resulting reads (Supplementary Table S1) were submitted to FastQC quality analysis and trimmed with Trimmomatic for all NEXTflex adaptors and barcodes. A total of 3 sets of *de novo* assembled transcriptomes were generated using Trinity (Trinity, RRID:SCR_013048) (Table 2); 1 set for each specimen was a pool of the 4 tissue samples to avoid assembly bias due to intraspecific polymorphism [16].

**Table 2:** Trinity assembled transcripts used in the assembly and annotation of *L. fortunei* genome

		Number of reads	Number of	Number of	Average	GC
Sample	Pooled tissues	prior assembly	trinity transcripts	trinity genes	contig length	%
Mussel 1	Gills, mantle, digestive gland, foot	406 589 144	433 197	303 172	854	34
Mussel 2	Gills, mantle, digestive gland, foot	376 577 660	435 054	298 117	824	34
Mussel 3	Gills, mantle, digestive gland, foot	334 316 116	499 392	351 649	844	34

### Genome assembly using a hybrid and hierarchical strategy

**Figure 1:**
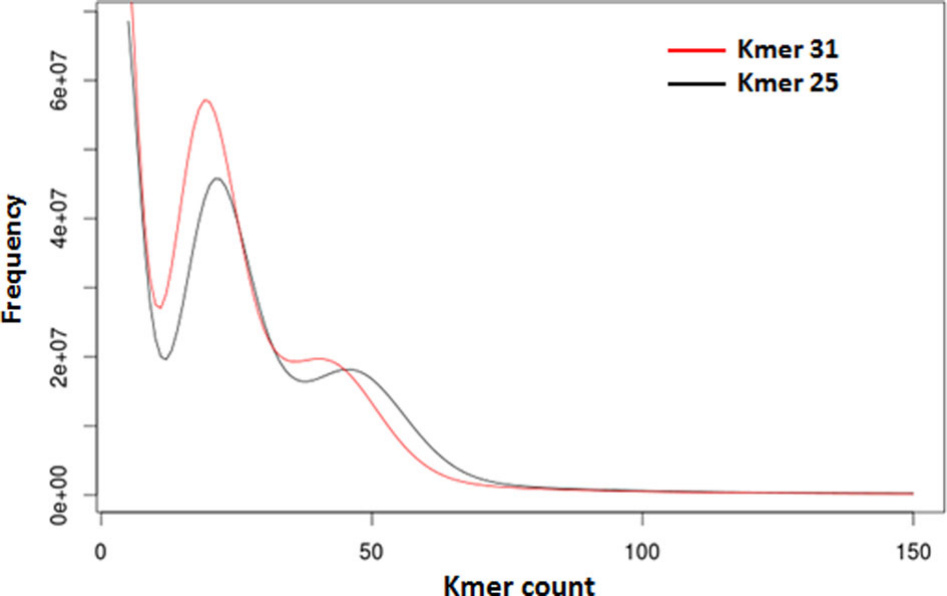
Kmer distribution of *Limnoperna fortunei* Illumina DNA reads (Table 1).

Jellyfish software (Jellyfish, RRID:SCR_005491) [17] was used to count and determine the distribution frequency of lengths 25 and 31 kmers (Fig. 1) for the Illumina DNA paired-end and mate-paired reads (Table 1). The genome size was estimated to be 1.6 Gb by using the 25-kmer distribution plot as total kmer number and then subtracting erroneous reads (starting kmer counts from ×12 coverage) to further divide by the homozygous coverage-peak depth (×45 coverage), as performed by Li et al. (2010) [18]. A double-peak kmer distribution was used as evidence of genome diploidy (Fig. 1) and high heterozygosity. The rate of heterozygosity was estimated to be 2.3%, and it was calculated as described by Vij et al. (2016) [19], using as input data the 17-kmer distribution plot for reads from 1 unique specimen.

**Figure 2:**
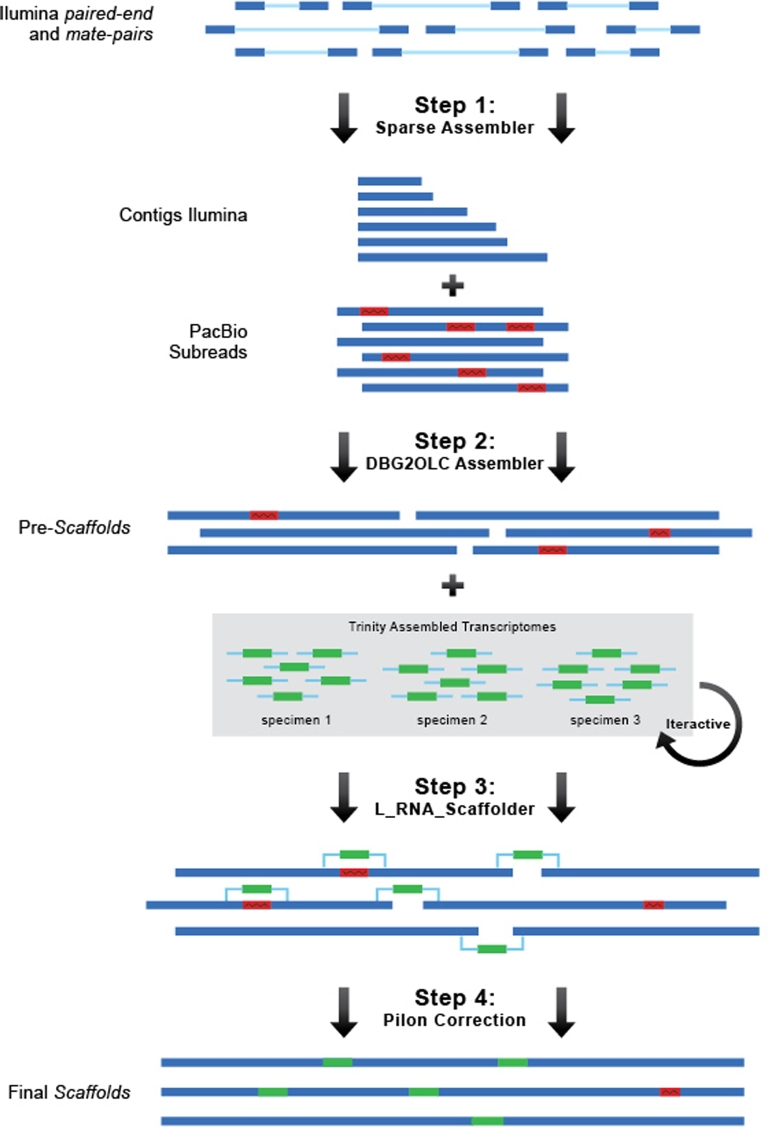
Hierarchical assembly strategy employed for the golden mussel genome assembly. Trimmed Illumina reads were assembled to the level of contigs with the Sparse Assembler algorithm (Step 1). Then, Illumina contigs and PacBio reads were used to build scaffolds with the DBG2OLC assembler, which anchors Illumina contigs to erroneous PacBio subreads, correcting them and building longer scaffolds (Step 2), followed by transcriptome joining scaffolds using L_RNA_scaffolder (Step 3). Final scaffolds were corrected by re-aligning all Illumina DNA and RNA-seq reads back to them and calling consensus with Pilon software (Step 4). In bold is the bioinformatics software used in each step. Red blocks indicate PacBio errors, which are represented by insertions and/or deletions, found in approximately 12% of PacBio subreads.

Initially, we attempted to assemble the golden mussel genome using only short Illumina reads of different insert sizes (paired-end and mate-paired) (Table 1) using traditional *de novo* assembly software such as ALLPATHS (ALLPATHS-LG, RRID:SCR_010742) [20], SOAPdenovo (SOAPdenovo, RRID:SCR_010752) [21], and MaSuRCA (MaSuRCA, RRID:SCR_010691) [22]. All these attempts resulted in very fragmented genome drafts, with an N50 no higher than 5 Kb and a total of 4 million scaffolds. To reduce fragmentation, we further sequenced additional long reads (10 PacBio SMTR Cells) (Table 1) and performed a hybrid and hierarchical *de novo* assembly, described below and depicted in Fig. 2.

First, (i) trimmed paired-end and mate-paired DNA Illumina reads (Table 1) were assembled into contigs using the software Sparse Assembler [23] with parameters *LD 0 NodeCovTh 1 EdgeCovTh 0 k 31 g 15 PathCovTh 100 GS 1 800 000 000*. Next, (ii) the resulting contigs were assembled into scaffolds using Pacific Biosciences long subreads data and the PacBio-correction-free assembly algorithm DBG2OLC [24] with parameters *LD1 0 k 17 KmerCovTh 10 MinOverlap 20 AdaptiveTh 0.01*. Finally, (iii) resulting scaffolds were submitted to 6 iterative runs of the program L_RNA_Scaffolder [25], which uses exon distance information from *de novo* assembled transcripts (Table 2) to fill gaps and connect scaffolds whenever appropriate. At the end, (iv) the final genome scaffolds were corrected for Illumina and Pacific Biosciences sequencing errors with the software PILON [26]: All DNA and RNA short Illumina reads were re-aligned back to the genome with BWA aligner (BWA, RRID:SCR_010910) [27], and resulting SAM files were BAM-converted, sorted, and indexed with the SAMTOOLs package (SAMTOOLS, RRID:SCR_002105) [28]. Pilon [26] identifies INDELS and mismatches by coverage of reads and yields a final corrected genome draft. Pilon was run with parameters *–diploid –duplicates.*

The final genome was assembled in 20 548 scaffolds, with an N50 of 312 Kb and a total assembly length of 1.6 Gb (Table 3).

**Table 3:** Assembly statistics for *Limnoperna fortunei*’s genome

Parameter	Value
Estimated genome size by kmer analysis, Gb	1.6
Total size of assembled genome, Gb	1.673
Number of scaffolds	20 548
Number of contigs	61 093
Scaffold N50, Kb	312
Maximum scaffold length, Mb	2.72
Percentage of genome in scaffolds >50 Kb	82.55
Masked percentage of total genome	33
Mapping percentage of Illumina reads back to scaffolds	91

The golden mussel genome presents 81% of all Benchmarking Universal Single Copy Orthologs (BUSCO version 3.3 analysis with Metazoa database; BUSCO, RRID:SCR_015008) (Table 4) and, compared with the mollusk genomes currently available [29–36], it represents one of the best assemblies of molluscan genomes so far also in terms of scaffold N50 and contiguity (Table 5).

**Table 4:** Summary statistics of BUSCO analysis for *L. fortunei* genome run for Metazoans

Categories	Number of genes	Percentage
Total BUSCO groups searched	978	–
Complete BUSCOs	801	81.9
Complete and single-copy BUSCOs	769	78.62
Complete and duplicated BUSCOs	32	3.27
Fragmented BUSCOs	72	7.36
Missing BUSCOs	105	10.73

**Table 5:** Comparison of genome assembly statistics for molluscan genomes

	*Haliotis*	*Lottia*	*Aplysia*	*Ruditapes*	*Patinopecten*	*Crassostrea*	**	*Mytillus*	*Bathymodiolus*	*Modiolus*	*Limnoperna*
	*discus hannai*	*gigantea*	*californica*	*philippinarum*	*yessoensis*	*gigas*	*Pinctadafucata*	*galloprovincialis*	*platifrons*	*philippinarum*	*fortunei*
Estimated genome size	1.65 Gb	359.5 Mb	1.8 Gb	1.37 Gb	1.43 Gb	545 Mb	1.15 Gb	1.6 Gb	1.64 Gb	2.38 Gb	1.6 Gb
Number of scaffolds	80 032	4475	8766	223 851	82 731	11 969	7997	1746 447	65 664	74 575	20 548
Total size of scaffolds	1 865 475 499	359 512 207	715 791 924	2 561 070 351	987 685 017	558 601 156	915 721 316	1 599 211 957	1 659 280 971	2 629 649 654	1 673 125 894
Longest scaffold	2 207 537	9 386 848	1 784 514	572 939	7 498 238	1 964 558	5 897 787	67 529	2 790 175	715 382	2 720 304
Shortest scaffold	854	1000	5001	500	200	100	1807	100	292	205	558
Number of scaffolds >1 K nt (%)	79 923 (99.9)	4471 (99.9)	8766 (100)	138 771 (61.9)	16 004 (19.3)	5788 (48.4)	7997 (100)	393 685 (22.5)	38 704 (58.9)	44 921 (60.2)	20 547 (100)
Number of scaffolds >1 M nt (%)	67 (0.1)	98 (2.2)	27 (0.3)	0 (0.0)	248 (0.3)	60 (0.5)	27 (0.3)	0 (0.0)	164 (0.2)	0 (0)	95 (0.5)
Mean scaffold size	23 309	80 338	81 655	11 441	11 939	46 671	114 508	916	25 269	35 262	81 425
Median scaffold size	1697	3622	13 763	1327	362	824	14 683	258	1284	13 722	22 134
N50 scaffold length	200 099	1 870 055	264 327	48 447	803 631	401 319	345 846	2651	343 373	100 161	312 020
Sequencing coverage	×322	×8.87	×11	×39.7	×297	×155	×234	×32	×319	×209.5	×60
Sequencing Technology	Illumina + PacBio	Sanger	Sanger	Illumina	Illumina	Illumina	Illumina + BACs	Illumina	Illumina	Illumina	Illumina + PacBio

One main challenges of assembling bivalve genomes lies in the high heterozygosity and amount of repetitive elements these organisms present: (i) the mussels *L. fortunei* and *Modiolus philippinarum* and the oyster *Crassostrea gigas* genomes were estimated to have heterozygosity rates of 2.3%, 2.02%, and 1.95%, respectively, which are substantially higher than other animal genomes [30], and (ii) repetitive elements correspond to at least 30% of the genomes of all studied bivalves so far (Table 3) [29–32, 34–36]. Also, retroelements might be active in some species such as *L. fortunei* (refer to the “Retroelements” section of this paper) and *C. gigas* [30], allowing genome rearrangements that may hinder genome assembly. One exception seems to be the deep-sea mussel *B. platifrons*, which has lower heterozygosity rates compared with other bivalves [32]. Sun et al. [32] suggested that it might be due to recurrent population bottlenecks that happened after events of population extinction and recolonization in the extreme environment [32]. Nevertheless, most of the bivalve genome projects relying only on short Illumina reads are likely to present fragmented initial drafts [29, 31]. PacBio long reads allowed us to increase the N50 to 32 Kb and to reduce the number of scaffolds from millions to 61 102, using the DBG2OLC [24] assembler. Finally, interactive runs of L_RNA_scaffolder [25] using the transcriptomes (Table 2) rendered the final result of N50 312 Kb in 20 548 scaffolds. It is important to note that assembly statistics can perform better for genomes assembled with reads generated with DNA extracted from 1 unique individual. This, however, was not possible for *L. fortunei*’s genome due to the high amount of high-quality DNA necessary to produce Illumina mate-pairs and PacBio long reads. In this study, the challenge of assembling the high polymorphic regions between haplotypes was enhanced by the difficulties of assembling reads that originated from highly polymorphic regions across individuals. However, the golden mussel assembly presented here shows that the use of Illumina contigs, low coverage of PacBio long reads, and transcriptome and Illumina re-mapping for final correction (Fig. 2) represent an option for cost-efficient assembly of highly heterozygous genomes of nonmodel species such as bivalves.

### Around 10% of repetitive elements are transposons

Initial masking of *L. fortunei* genome was done using the RepeatMasker program (RepeatMasker, RRID:SCR_012954) [37] with the parameter *-species bivalves* and masked 3.4% of the total genome. This content was much lower than the masked portion of other molluscan genomes, 34% in *C. gigas* [30] and 36% in *M. galloprovincialis* [29], suggesting that the fast evolution of interspersed elements limits the use of repeat libraries from divergent taxa [38]. Thus, we generated a *de novo* repeat library for *L. fortunei* using the program RepeatModeler (RepeatModeler, RRID:SCR_015027) [39] and its integrated tools RECON [40], TRF [41], and RepeatScout [42]. This *de novo* repeat library was the input to RepeatMasker, together with the first masked genome draft of *L. fortunei*, and resulted in a final masking of 33.4% of the genome. Even though more than 90% of the repeats were not classified by RepeatMasker (Supplementary Table S2), 8.85% of the repeats were classified as LINEs, Class I transposable elements. In addition, large numbers of reverse-transcriptases (824 counts, Pfam RVT_1 PF00078), transposases (177 counts, Pfam HTH_Tnp_Tc3_2 PF01498), integrases (501 counts, Pfam Retroviral integrase core domain PF00665), and other related elements were detected; more than 98% of these had detectable transcripts.

#### More than 30 000 sequences were identified by gene prediction and automated annotation

To annotate the golden mussel genome, we sequenced a number of transcriptomes (Table S1), *de novo* assembled (Table 2) and aligned these transcriptomes to the genome scaffolds, and created gene models with the PASA pipeline [37]. These models were used to train and run the *ab initio* gene predictor AUGUSTUS (Augustus: Gene Prediction, RRID:SCR_008417) (Supplementary Fig. S1) [38]. The complete gene models yielded by PASA [43] were BLASTed (e-value 1e-20) against the Uniprot database (UniProt, RRID:SCR_002380), and those with 90% or more of their sequences showing in the BLAST hit alignment were considered for further analysis. Next, all the necessary filters to run an AUGUSTUS [44] personalized training were performed: (i) only gene models with more than 3 exons were maintained, (ii) sequences with 90% or more overlap were withdrawn and only the longest sequences were retained, and (iii) only gene models free of repeat regions, as indicated by BLASTN similarity searches with *de novo* library of repeats, were maintained. These curated data yielded a final set of 1721 gene models on which AUGUSTUS [36] was trained in order to predict genes in the genome using the default AUGUSTUS [44] parameters. Once the gene models were predicted, a final step was performed by using the PASA pipeline [43] once again in the *update* mode (parameters -c -A -g -t). This final step compared the 55 638 gene models predicted by AUGUSTUS [44] with the 40 780 initial transcript-based gene-structure models from PASA [43] to generate the final set of 60 717 gene models for *L. fortunei.* Of those, 58% had transcriptional evidence based on RNA Illumina reads (Table S2) re-mapping, a rate that was expected as our RNA-Seq libraries were constructed for only 4 tissues of adult golden mussel specimens without any environmental stress induction (Table 2). Therefore, these libraries lack transcripts for developmental stages for some other cell types (i.e., hemocytes) and stress-inducible genes. Finally, 67% of the gene models were annotated by homology searches against Uniprot or NCBI NR (Table 6).

**Table 6:** Summary of gene annotation against various databases for *L. fortunei* whole-genome-predicted genes

Total number of genes	60 717
Total number of exons	220 058
Total number of proteins	60 717
Average protein size, aa	304
Number of protein BLAST hits* with Uniprot	26 198
Number of protein BLAST hits* with NR NCBI (no hits with Uniprot)	14 810
Number of protein HMMER hits* with Pfam.A	24 513
Number with proteins with KO assigned by KEGG	8387
Number of proteins with BLAST hits* with EggNOG	36 868

*All considered hits had a minimum e-value of 1e-05.

#### Protein clustering indicates evolutionary proximity among mollusk species

**Figure 3:**
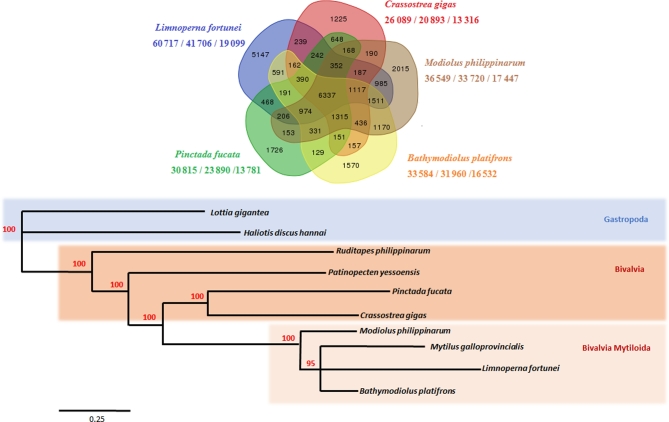
(A) Gene family assigned with OrthoMCL for the total set of proteins predicted from 5 mussel genome projects. Outside the Venn diagram, the species name is represented, and below it is the number of proteins/number of clustered proteins/number of clusters. (B) Phylogeny of the concatenated dataset using 44 single-copy orthologs extracted from 10 molluscan genomes. The VT model was estimated to be the best-fitting substitution model with ProtTest 3.4.2. We reconstructed the phylogeny using PhyML and 100 bootstrap repetition.

Gene family relationships were assigned using reciprocal best BLAST and OrthoMCL software (version 1.4) [45] between *L. fortunei* proteins and the total protein set predicted for 9 other mollusks: the mussels *M. galloprovincialis, M. philippinarum*, and *B. platifrons*, the clam *Ruditapes philippinarum*, the scallop *Patinopecten yessoensis*, the pacific oyster *C. gigas*, the pearl oyster *Pinctada fucata* (genome version from Du et al. [36]), and the gastropods *Lottia gigantea* and *Haliotis discus hannai* (see Supplementary Table S3 for detailed information on the comparative data). Figure 3A presents orthologs relationships for 5 of the bivalves analyzed. A total of 6337 ortholog groups are shared among the 5 bivalve species.

Of all the orthologs found for the total 10 species, 44 groups are composed of single-copy orthologs containing 1 representative protein sequence of each species. These sequences were used to reconstruct a phylogeny: the single-copy ortholog sequences were concatenated and aligned with CLUSTALW [46], with a resulting alignment 30 755 sites in length (Fig. 3B). ProtTest 3.4.2 [47] was used to estimate the best-fitting substitution model, which was VT+G+I+F [48]. With this alignment and model, we reconstructed the phylogeny using PhyML [49] and 100 bootstrap repetition; the resulting tree is shown in Fig. 3B.

#### Protein domain analysis shows expansion of binding domain in L. fortunei

We performed a quantitative comparison of protein domains predicted from whole-genome projects of 10 molluscan species. The complete protein sets of *M. galloprovincialis, M. philippinarum, B. platifrons, Ruditapes philippinarum, Patinopecten yessoensis, C. gigas, Pinctada fucata, Lottia gigantean*, and *Haliotis discus hannai* (Supplementary Table S3) were submitted to domain annotation using HMMER against the Pfam-A database (e-value 1e-05). Protein expansions in *L. fortunei* were rendered using the normalized Pfam count value (average) obtained from the other 9 mollusks, according to a model based on the Poisson cumulative distribution. Bonferroni correction (*P* ≤ 0.05) was applied for false discovery, and absolute frequencies of Pfam-assigned domains were initially normalized by the total count number of Pfam-assigned domains found in *L. fortunei* to compensate for discrepancies in genome size and annotation bias.

**Figure 4:**
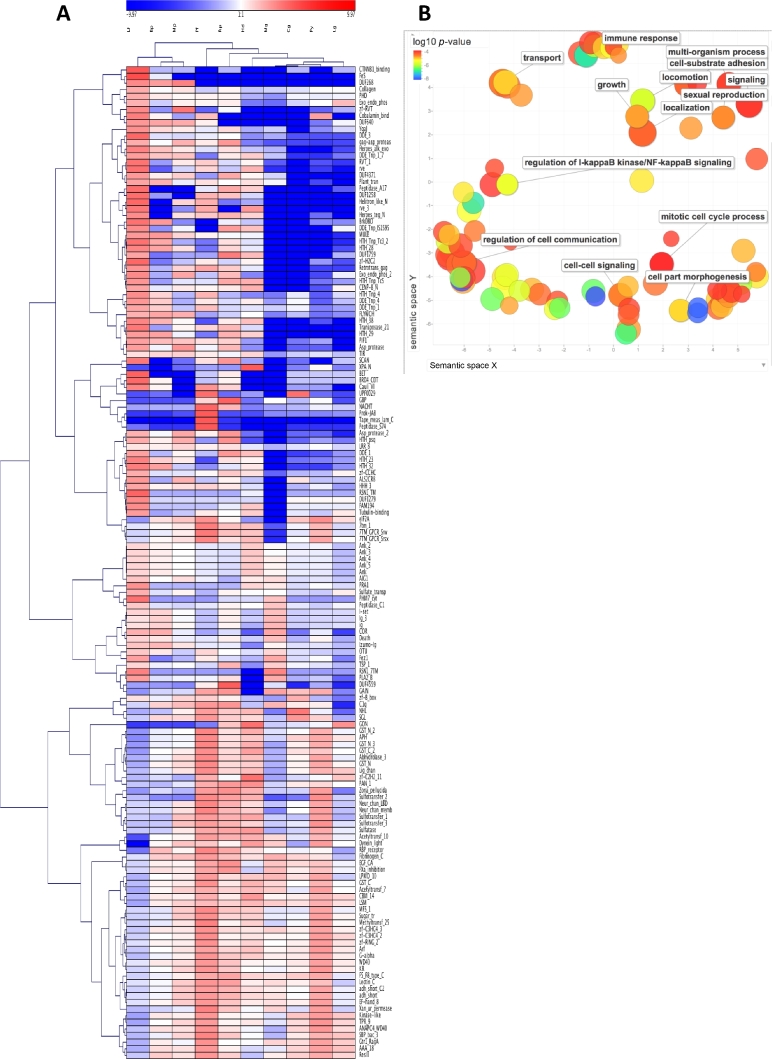
Gene family representation analysis in the *L. fortunei* genome. (A) Pfam hierarchical clustering, heatmap. Features were selected according to a model based on the Poisson cumulative distribution of each Pfam count in the golden mussel genome vs the normalized average values found in the other 9 molluscan genomes (Bonferroni correction, *P* ≤ 0.05). Transposable elements were included in the analysis. Colors depict the log2 ratio between Pfam counts found in each single genome and the corresponding mean values. The hierarchical clustering used the average dot product for the data matrix and complete linkage for branching. Abbreviations: Bp: *Bathymodioulus platifrons*; Cg: *Crassostrea gigas*; Hd: *Haliotus discus hannai*; Lf: *L. fortunei*; Lg: *Lottia gigantean*; Mg: *Mytilus galloprovincialis*; Mp: *Modioulus philippinarum*; Pf: *Pinctada fucata*; Py: *Patinopecten yessoensis*; Rp: *Ruditapes philippinarum*. (B) Gene Ontology analysis of expanded gene families, semantic scatter plot. Shown are cluster representatives after redundancy reduction in a 2-dimensional space applying multidimensional scaling to a matrix of semantic similarities of GO terms. Color indicates the GO enrichment level (legend in upper left-hand corner); size indicates the relative frequency of each term in the UNIPROT database (larger bubbles represent less specific processes).

For *L. fortunei*, the annotation against Pfam-A classified 40 127 domains in 24 513 gene models, of which 83 and 67 were expanded or contracted, respectively, in comparison with the other mollusks (Fig. 4A; Supplementary Table S4 and S5). The 83 overrepresented domains were further analyzed for functional enrichment using domain-centric Gene Ontology (Fig. 4B). The analysis shows a prominent expansion of binding domains in *L. fortunei*, such as Thrombospondin (TSP_1), Collagen, Immunoglobulins (Ig, I-set, Izumo-Ig Ig_3), and Ankyrins (Ank_2, Ank_3, and Ank_4). These repeats have a variety of binding properties and are involved in cell-cell, protein-protein, and receptor-ligand interactions driving the evolutionary improvement of complex tissues and the immune defense system in metazoans [50–54]. An evolutionary pressure toward the development of a diversified innate immune system is also suggested by the high amount of leucine rich repeats (LRR) and Toll/interleukin-1 receptor homology domains (TIR). Death, another over-represented Pfam, is also part of TLR signaling, being present in several docking proteins such as Myd88, Irak4, and Pelle [55]. Interestingly, BLAST analysis of *L. fortunei* gene models against Uniprot identified 2 types of TLRs whose prototypical architecture of N-terminal extracellular LRR motifs including either a single or multiple cysteine cluster domain, a C-terminal TIR domain spaced by a single transmembrane-spanning domain [56], could be correctly identified using the Simple Modular Architecture Research Tool (SMART) [57]. Indeed, we confirmed 141 sequences with similarity to single cysteine clusters TLRs (scc) typical of vertebrates and 29 sequence hits with the multiple cysteine cluster TLRs (mcc) typical of *Drosophila* [56]. Phylogenetic analysis of all sequences (using PhyML [49], model JTT) (Supplementary Fig. S2) shows evidence for TLRs clade separation in *L. fortunei*; the scc TLRs exhibit a higher degree of amino acid changes, higher molecular evolution, and diversification than the mcc TLRs. Overall, the expansion of these gene families might suggest an improved resistance to infections. It is, however, equally curious that other immune-related gene families such as Fribinogen_C and C1q seem to be contracted (Supplementary Table S5). This feature may depend on the evolution-driven, yet random fate of the *L. fortunei* genome, a consequence of different specific duplicate genes in other species. Also, other protein families involved in toxin metabolism, especially glutathione-based processes and sulfotransferases, are clearly contracted (Table S5).

### Final considerations

Here we have described the first version of the golden mussel complete genome and its automated gene prediction, which were funded through a crowdfunding initiative in Brazil. This genome contains valuable information for further evolutionary studies of bivalves and metazoa in general. Additionally, our team will further search for the presence of proteins of biotechnology interest such as the adhesive proteins produced by the foot gland that we have described elsewhere [58] or genes related to the reproductive system that have been shown to be very effective for invertebrate plague control [59]. The golden mussel genome and the predicted proteins are available for download in the GigaScience repository, and the scientific community is welcome to further curate the gene predictions.

As the golden mussel advances towards the Amazon River Basin, the information provided in this study may be used to help develop biotechnological strategies that may control the expansion of this organism in both industrial facilities and open environment.

## Availability of supporting data

*Limnoperna fortunei's* genome and transcriptome data are available in the Sequence Read Archive (SRA) as BioProject PRJNA330677 and under the accession numbers SRR5188384, SRR5195098, SRR518800, SRR5195097, SRR5188315, SRR5181514. This Whole Genome Shotgun Project has been deposited in the DDBJ/ENA/GenBank under accession number NFUK00000000. The version described in this paper is version NFUK01000000. Supporting data, also including annotations and BUSCO results, are available via the *GigaScience* repository, *Giga*DB [60].

## Additional files

Supplementary Table S1. RNA raw reads sequenced for 3 *L. fortunei* specimens, 4 tissues each.

Supplementary Table S2. RepeatMasker classification of repeats predicted in the *L. fortunei* genome.

Supplementary Table S3. Details of the online availability of the data used for ortholog assignment and protein domain expansion analysis.

Supplementary Table S4. Expanded protein families in the *L. fortunei* genome.

Supplementary Table S5. Contracted protein families in the L. *fortunei* genome.

Supplementary Table S6. Fantasy names given to *L. fortunei* genes and proteins from the backers that supported us through crowdfunding (www.catarse.me/genoma).

Supplementary Figure 1. Steps performed for the prediction and annotation of the *L. fortunei* genome.

Supplementary Figure 2. Phylogenetic tree of Toll-like (TLRs) receptors found in the *L. fortunei* genome.

## Abbreviations

BUSCO: Benchmarking Universal Single-Copy Orthologs; KEGG: Kyoto Encyclopedia of Genes and Genomes; SRA: Sequence Read Archive.

## Ethics approval

*Limnoperna fortunei* specimens used for DNA extraction and sequencing were collected in the Jacuí River (29°59΄29.3″S 51°16΄24.0″W), southern Brazil. This bivalve is an exotic species in Brazil and is not characterized as an endangered or protected species.

## Competing interests

The authors declare that they have no competing interests.

## Funding

This work was supported by the Brazilian Government agencies CAPES (PVE 71/2013), FAPERJ APQ1 (2014), and FAPERJ/DFG (39/2014). Also, this work was funded through crowdfunding with the support of 346 people (www.catarse.me/genoma).

## Author contributions

Conceived and designed the experiments: M.R., M.U., T.O., C.M., F.D. Performed the experiments: M.U., J.A. Analyzed the data: M.U., T.O., C.M., F.D., F.P., N.C., I.C., M.R. Contributed reagents/materials/analysis tools: M.R., F.P., C.M. Wrote the paper: M.U., F.D., M.R. All authors read and approved the final manuscript.

## Supplementary Material

GIGA-D-17-00124_Original_Submission.pdf

GIGA-D-17-00124_Revision_1.pdf

GIGA-D-17-00124_Revision_2.pdf

Response_to_Reviewer_Comments_Original_Submission.pdf

Response_to_Reviewer_Comments_Revision_1.pdf

Reviewer_1_Report_(Original_Submission) -- Marco Gerdol 31 Jul 2017 Reviewed

Reviewer_1_Report_(Revision_1) -- Marco Gerdol 08 Nov 2017 Reviewed

Reviewer_2_Report_(Original_Submission) -- Kevin Kocot 23 Aug 2017 Reviewed

Supplemental material
