# Supplementary material for: A hybrid-hierarchical genome assembly strategy to sequence the invasive golden mussel, Limnoperna fortunei
Source: Gigascience. 2017 Dec 15;7(2):gix128. doi: 10.1093/gigascience/gix128 (PMC5836269; doi:10.1093/gigascience/gix128)
Supplement: GIGA-D-17-00124_Revision_1.pdf [file gix128_giga-d-17-00124_revision_1.pdf]

GigaScience

A hybrid-hierarchical genome assembly strategy to sequence the invasive golden mussel Limnoperna fortunei

--Manuscript Draft--

**Manuscript Number:**	GIGA-D-17-00124R1
**Full Title:**	A hybrid-hierarchical genome assembly strategy to sequence the invasive golden mussel Limnoperna fortunei
**Article Type:**	Data Note
**Funding Information:**	CAPES (PVE (71/2013)) — Dr Mauro F Rebelo<br>FAPERJ (APQ1 (2014)) — Dr Mauro F Rebelo<br>FAPERJ/DFG (FAPERJ/DFG (39/2014)) — Dr Mauro F Rebelo<br>Crowdfunding (www.catarse.me/genoma) — Dr Mauro F Rebelo
**Abstract:**	Background: For more than 25 years, the golden mussel Limnoperna fortunei has aggressively invaded South American freshwaters, having travelled more than 5,000 km upstream across five countries. Along the way, the golden mussel has outcompeted native species and economically harmed aquaculture, hydroelectric powers, and ship transit. We have sequenced the complete genome of the golden mussel to understand the molecular basis of its invasiveness and search for ways to control it. Findings: We assembled the 1.6 Gb genome into 20548 scaffolds with an N50 length of 312 Kb using a hybrid and hierarchical assembly strategy from short and long DNA reads and transcriptomes. A total of 60717 coding genes were inferred from a customized transcriptome-trained AUGUSTUS run. We also compared predicted protein sets with those of complete molluscan genomes, revealing an exacerbation of protein-binding domains in L. fortunei. Conclusions: We built one of the best bivalve genome assemblies available using a cost-effective approach using Illumina pair-end, mate pair, and PacBio long reads. We expect that the continuous and careful annotation of L. fortunei's genome will contribute to the investigation of bivalve genetics, evolution, and invasiveness, as well as to the development of biotechnological tools for aquatic pest control.
**Corresponding Author:**	Marcela Uliano da Silva, Ph.D<br>Universidade Federal do Rio de Janeiro<br>Rio de Janeiro, RJ BRAZIL
**Corresponding Author Secondary Information:**	
**Corresponding Author's Institution:**	Universidade Federal do Rio de Janeiro
**Corresponding Author's Secondary Institution:**	
**First Author:**	Marcela Uliano da Silva, Ph.D
**First Author Secondary Information:**	
**Order of Authors:**	Marcela Uliano da Silva, Ph.D<br>Francesco Dondero, Ph.D<br>Thomas D Otto, Ph.D<br>Igor R Costa, Msc<br>Nicholas CB Lima, Ph.D<br>Juliana A Americo, Ph.D<br>Camila J Mazzoni, Ph.D

Powered by Editorial Manager® and ProduXion Manager® from Aries Systems Corporation

	Francisco Prosdocimi, Ph.D
	Mauro F Rebelo, Ph.D
**Order of Authors Secondary Information:**	
**Response to Reviewers:**	We thank the reviewers for their attentive read of the manuscript and for suggesting revisions that have increased the overall quality of the data presentation and of the manuscript. Please find bellow each reviewers comment and the answers to them:<br><br>Reviewers 1:<br><br>Reviewer 1 - Line 49: could the authors provide an extended background to the readers about the arrival of this invasive species in South America?<br><br>Response: Yes, the extended background was provided and it's situated in lines 53-55 in the new submission. It is as follows: "... Research suggests that L. fortunei was introduced in South America through ballast water of ships coming from Hong Kong or Korea [2]. It was found for the first time in the estuary of the La Plata River in 1991 [1]."<br><br>Reviewer 1 - Line 66: it is maybe better to specify here "freshwater bivalves". Indeed, many other species could be considered as "invasive" in the marine environment, including Mytilus spp.<br><br>Response: "Freshwater" was added at line 72.<br><br>Reviewer 1 - Line 76: Also, L. fortunei is a mytiloid and other mussel species are known to display an exceptional tolerance to biotic and abiotic contamination, with remarkable capabilities of accumulation and metabolization of toxicants. It is possible that golden mussels share some of these features with marine mussels.<br><br>Response: It's true. But we kept the introduction as it was in order to keep it concise and cohesive.<br><br>Reviewer 1 - *Lines 96-97: The choice to use three mussels for DNA extraction and sequencing is unclear (unless this is a typo related to the use of 3 mussels for RNA extraction). Why did the authors choose to use this non-standard procedure? Was the genomic DNA extracted from three different specimens pooled in equimolar quantities and used for sequencing? Usually, as heterozygosity might represent a considerable issue, it is desirable to use a single specimen as a reference for genome assembly.<br><br>Response: The idea was to sequence only one specimen. But it was not possible due to (i) Illumina DNA library preparation unanticipated problems and (ii) the amount of DNA necessary for PacBio sequencing. The sequencing facility responsible for producing Illumina pair-end and mate pair reads (UNESP) failed to produce the mate pairs in their first attempt, and they asked for more DNA to repeat the library preparation. As we did not have more tissue from the first specimen, we needed to extract more from a second specimen. After that, as we notice the use of only Illumina would not allow us to produce a contiguous high-quality genome, we decided to sequence PacBio. PacBio libraries need a substantial amount of high-molecular-weight-DNA, and to meet this requirements we needed to extract DNA from a third specimen.<br><br>To clarify the use of 3 specimens for the construction of the 3 sequencing libraries, a small complement was added to the sentence in line 103-105. It's as follows "... For the genome assembly, a total of 3 individuals were sampled for DNA extraction from gills and to produce the three types of DNA libraries used in this study."<br><br><br>Reviewer 1 - Lines 137-138: Please indicate what the two colors in figure 1 correspond to (I guess to two different k-mer length, but this is not specified neither in the figure itself, nor in its caption. Also, the relative size of the heterozygous peak compared to the homozygous one is particularly remarkable and indicates an extremely high heterozygosity rate, which the authors could estimate and report. This could be linked easily with the subsequent paragraph and the difficulties in assembling such a highly

Powered by Editorial Manager® and ProduXion Manager® from Aries Systems Corporation

heterozygous genome using short reads only. Please note that these issues have been also encountered by Murgarella and colleagues in the draft assembly of the M. galloprovincialis genome.

Response: We have added the legend on the figures representing the colors. Red represented a the distribution of kmers size 31 and black represented the kmers of size 25. Also, we have estimated the heterozygosity rate of L. fortunei genome to be 2.07%, and we have included this information and some comments between the lines 150-152 It is as follows: “The rate of heterozygosity was estimated to be 2.07% and it was calculated as described by Vij et al. (2016) [18], using as input data the 25-kmer distribution plot for reads from one unique specimen”.

And also we did some editings in lines 185-190 . It is as follows “...One main challenge of assembling bivalve genomes lies in the high heterozygosity and amount of repetitive elements these organisms present: (i) the mussels L. fortunei and Modiolus philippinarum and the oyster Crassostrea gigas genomes were estimated to have heterozygosity rates of 2.07%, 2.02 % 1.95% respectively, which is substantially higher than other animal genomes [29], and (ii) repetitive elements correspond to at least 30% of the genomes of all studied bivalves so far (Table 3) [28, 29, 30, 31, 33, 34, 35]. “

Reviewer 1 - *Table 5 and Figure 3 would benefit from the inclusion of a few recently released genomes of other bivalves. Specifically, a much improved version of the Pinctada fucata genome has just been released on Gigascience (the authors could not have access to this resource at the time of writing their manuscript): https://academic.oup.com/gigascience/article/4034775/The-pearl-oyster-Pinctada-fucata-martensii-genome?searchresult=1.
At the same time, the genome of the pectinoid Mizuhopecten yessoensis has also been released (data is available at http://mgb.ouc.edu.cn/pydatabase/download.php).
The genome of the veneroid clam Ruditapes philippinarum is also now available: https://academic.oup.com/gbe/article-lookup/doi/10.1093/gbe/evx096
In this case, while sequence data is not publicly available yet, the authors are willing to share their data upon request.

Response: The 3 new bivalve genomes (P. fucata, M. yessoensis and R. philippinaum) were included in all the comparative analysis of this paper: in Table 3 and Figures 3 and 4. The previous P. fucata data was replaced, and now comparisons were done with the new assembly presented by Du et al (https://doi.org/10.1093/gigascience/gix059). Table S3 was updated accordingly. And also line 272.

Reviewer 1 - Line 235: "these genomes" should be "these transcriptomes"
Response: It was corrected. Line 234.

Reviewer 1 - Line 251: the authors could add a brief comment about the 58% rate of gene whose expression could be confirmed, stating that this is a reasonable and even expected result, based on the absence of libraries gathered from developmental stages, some adult tissues (i.e. hemocytes) and mussels subjected to different stress (so that inducible gene products might be absent).

Response: The comment was introduced in line 250-255: It is as follows “...Of those, 58% had transcriptional evidence based on RNA Illumina reads (Table S2) re-mapping, rate that was expected since our RNA-Seq libraries were constructed only for 4 tissues of adult golden mussel specimens without any environmental stresses induction (Table 2). Therefore, these libraries lack transcripts for developmental stages, for some other cell types (i.e. hemocytes) and stress-inducible genes. Finally, 67% of the gene models were annotated by homology searches against Uniprot or NCBI NR (Table 6).”

Reviewer 1 - Lines 27-273: "five mussels" should be "five bivalves". Also, this data could be updated using the newly released bivalve genomes I have listed above.

Powered by Editorial Manager® and ProduXion Manager® from Aries Systems Corporation

Response: This was corrected and the information, Supl Table S3, and Figure 3 were updated with the new species included in the analysis. Lines 275.

Reviewer 1 - *Line 276: "reconstruct phylogeny" needs to be detailed. What strategy was used (Bayesian, ML, NJ?), what model of molecular evolution, what software? Are the support values displayed in the tree posterior probabilities or bootstrap values?

Response: The methods used were more detailed in lines 277-282. Also, the updated phylogeny was performed including the new data for the P. fucata genome, replacing the old one used, and also including the new data recommended by the review for R. phillapirum and P. yeoensis. It is as follows: " These sequences were used to reconstruct a phylogeny: the single-copy orthologs sequences were concatenated and aligned with CLUSTALW [45] with a resulting alignment of 30755 sites in length (Figure 3B). ProtTest 3.4.2 [46] was used to estimate the best fitting substitution model, which was VT [47]. With this alignment and model we reconstructed the phylogeny using PhyML [48] and 100 bootstrap repetition, the resulting tree is shown on Figure 3B."
.

Reviewer 1 - *Line 301: TIR domains do not necessarily belong to TLRs. More than half of bivalve TIR-DC proteins are indeed intracellular receptors of unknown function (but which are still likely involved in intracellular immune signaling (see Gerdol et al, DCI 2017). The interpretation of Figure S2 and the discussion contained in lines 303-309 is therefore quite difficult to be evaluated without knowing whether only proteins containing LRRs+TIR or all those containing TIR domains (with and without LRRs) were taken into account. Furthermore, BLAST is not overly useful, by itself, to classify these proteins, as it has been previously demonstrated.
Considering the complexity of this topic and the fact that this goes probably beyond the scopes of this manuscript, the authors could simplify tis section by reporting and expanded complement of TIR-DC proteins and DEATH-domain containing proteins of different nature which, accordingly to the know functions of these domain and existing literature data, are likely to be involved in immune signaling. Overall the expansion of these gene families might suggest an improved resistance to infections. It is however equally curious that other immune-related gene families (e.g. FREPs and C1qDC) seem to be somewhat contracted in figure 4.

Response: Having found LRRs and TIR in the list of over-represented PFAM we looked for TLRs in Blast results, since it was logical to find many of them. However, we were completely aware that not all those Blast hits could represent a genuine TLR, since Blast is heuristically biased towards short High Scoring Pairs (HSP) that could be tagged only to a TIR domain. We, therefore, used SMART (Simple Modular Architecture Research Tool, see http://smart.embl-heidelberg.de/help/smart_about.shtml) to analyze all Blast TLR hits for their modular domain architectures. Only those sequences showing a prototypical TLR architecture were further considered, i.e. N-terminal extracellular leucine-rich repeat (LRR) motifs including either a single or multiple cysteine cluster domain, a C-terminal TIR domain spaced by a single transmembrane-spanning domain (Leulier & Lemaitre, 2008). We know this analysis is not conclusive but TLR expansions in lophotrochozoa were not known until a few years ago when it has been demonstrated in anellida. This finding can contribute to stimulate TLR evolutionary studies. We added some details of the analysis in the body text to explain that those TLR we considered are representative of genuine TLRs.

We have changed a few sentences in the manuscript accordingly. Lines 319-325: It is as follows: "Overall, the expansion of these gene families might suggest an improved resistance to infections. It is, however, equally curious that other immune-related gene families such as Fribinogen_C and C1q seem to be contracted (Supplementary Table S5). This feature may depend on the evolutionary-driven, yet random, fate of the L. fortunei genome and consequence of different specific duplicate genes in other species. Also, other protein families involved in toxin metabolism, especially glutathione based processes and sulfotransferases are clearly contracted (Table S5)."

Powered by Editorial Manager® and ProduXion Manager® from Aries Systems Corporation

Reviewer 1 - Line 555: bellow -> below
Response: Thank you, it was corrected. Line 611.

Reviewer 1 - *In Figure 4 legend, it is specified that transposable elements were taken into account. I guess that, depending on the annotation pipeline followed by the different genome sequencing projects these might have been either masked or not, thereby being often excluded from the final protein set. While the heat map seems to show that TEs are, in general, extremely expanded in Limnoperna, I would be very careful about this claim. This also applies to Table S4. Considering the very high number of gene predictions corresponding to TEs in Limnoperna a particular attention should be also posed into the calculations of under-representation of domains, as these were made based on relative abundance, which would be de facto lowered in Limnoperna if TEs have been masked in the other molluscan genomes.

Response: We agree with this comment, and it was, in fact, a relevant debate among us if we should include or not such retro-domains in the analysis. However, as it seems that such sequences can have a central biological role in shaping some L fortunei genomic features (and maybe physiological ones), we decided to show them even knowing that in other genome studies they might have been kept out or not considered with attention. Indeed, some genomes we used for the new comparison presented in this revised ms, did include TEs in their annotation analysis, e.g. Ruditapes philippinarum, Haliotis discus, Modiolus philippinarum (See Table 5 of the revised ms). The golden mussel genome always outperformed these numbers. However, we tested how considering TE elements in our PFAM analysis might have biased the down-represented features. The reviewer comment has been very appropriate since it can happen and we were not aware of that. Nevertheless, we are confident of the genuinity of our analysis and results. In fact, we made some trials considering a lower total PFAM count value for frequency normalization in other mollusc genomes. When we re-normalized PFAM frequencies at 5% or 10% less counts than before, about 25% and 50% PFAMs are excluded from the original list. Considering that (i) we have estimated about 2500 PFAM countss (nearly 6%); (ii) some other annotations included in the analysis are actually using PFAM associated to TEs; (iii) we used the most conservative false discovery rate procedure, i.e. Bonferroni's; we can conclude that excluding TE from this analysis can be more detrimental than beneficial to the correct functional annotation of the golden mussel genome.

Reviewer 1 - Table S3: "4 other mollusk" -> please correct 4
Response: Table S3 was updated.

Reviewer #2 (Kevin Kocot): Specific comments:
There are too many very short paragraphs. A paragraph should always have at least two sentences. The paragraph spanning lines 58-65 covers two disparate topics and the introduction of the text may need to be reorganized.

Response: we tried to avoid the short paragraph as much as possible. For example, adding a short paragraph to the last line of Table 3, and then deleting it from the manuscript.

Reviewer 2: Why were multiple individuals used?

Response: The idea was to sequence only one specimen. But it was not possible due to (i) Illumina DNA library preparation unanticipated problems and (ii) the amount of DNA necessary for PacBio sequencing. The sequencing facility responsible for producing Illumina pair-end and mate pair reads (UNESP) failed to produce the mate pairs in their first attempt, and they asked for more DNA to repeat the library preparation. As we did not have more tissue from the first specimen, we needed to extract more from a second specimen. After that, as we notice the use of only Illumina would not allow us to produce a contiguous high-quality genome, we decided to sequence PacBio. PacBio libraries need a substantial amount of high-molecular-weight-DNA, and to meet this requirements we needed to extract DNA from a third specimen.

To clarify the use of 3 specimens for the construction of the 3 sequencing libraries, a small complement was added to the sentence in line 103-105. It's as follows "... For the

Powered by Editorial Manager® and ProduXion Manager® from Aries Systems Corporation

	genome assembly, a total of 3 individuals were sampled for DNA extraction from gills and to produce the three types of DNA libraries used in this study."<br><br>Reviewer 2 : The recent Crown of Thorns sea star genome paper (http://www.nature.com/nature/journal/v544/n7649/full/nature22033.html?foxtrotcallback=true) would be an appropriate citation on line 82.<br>Response: The citation was added. It's now present in line 88.<br><br>Reviewer 2: Line 85: Change "U$ " to "USD $"<br>Response: It was changed in line 91.<br><br>Reviewer 2: Lines 166-167: I suggest the authors move this text to the table.<br>Response: The small paragraph was removed and now it is presented as the last line of Table 3.<br><br>Lines 266-273: Despite the name, OrthoMCL does not identify orthologs, it identifies gene families. These are gene family comparisons and not strict orthologs.<br><br>Response: Manuscript was edited. Line 268.
**Additional Information:**	
**Question**	**Response**
Are you submitting this manuscript to a special series or article collection?	No
**Experimental design and statistics**<br><br>Full details of the experimental design and statistical methods used should be given in the Methods section, as detailed in our Minimum Standards Reporting Checklist. Information essential to interpreting the data presented should be made available in the figure legends.<br><br>Have you included all the information requested in your manuscript?	Yes
**Resources**<br><br>A description of all resources used, including antibodies, cell lines, animals and software tools, with enough information to allow them to be uniquely identified, should be included in the Methods section. Authors are strongly encouraged to cite Research Resource Identifiers (RRIDs) for antibodies, model organisms and tools, where possible.<br><br>Have you included the information requested as detailed in our Minimum Standards Reporting Checklist?	Yes
**Availability of data and materials**	Yes

All datasets and code on which the conclusions of the paper rely must be either included in your submission or deposited in publicly available repositories (where available and ethically appropriate), referencing such data using a unique identifier in the references and in the “Availability of Data and Materials” section of your manuscript.<br><br>Have you have met the above requirement as detailed in our Minimum Standards Reporting Checklist?	

Powered by Editorial Manager® and ProduXion Manager® from Aries Systems Corporation

Manuscript Click here to download Manuscript UlianoSilv-GoldenMussel-revised.docx

DATA NOTE

A hybrid-hierarchical genome assembly strategy to sequence the invasive golden mussel *Limnoperna fortunei*

Authors: Marcela Uliano-Silva[1,2,3*], Francesco Dondero[4], Thomas Dan Otto[5,6], Igor Costa[7], Nicholas Costa Barroso Lima[7,8], Juliana Alves Americo[1], Camila Junqueira Mazzoni[2,3], Francisco Prosdocimi[7], Mauro de Freitas Rebelo[1*]

Marcela Uliano-Silva: marcela.uliano@gmail.com
Francesco Dondero: francesco.dondero@uniupo.it
Thomas D. Otto: tdo@sanger.ac.uk
Igor Costa: igor.bioinfo@gmail.com
Nicholas Costa Barroso Lima: ncblima@gmail.com
Juliana Alves Americo: juliana.americo@gmail.com
Camila Mazzoni: mazzoni@izw-berlin.de
Francisco Prosdocimi: prosdocimi@bioqmed.ufrj.br
Mauro de Freitas Rebelo: mrebelo@biof.ufrj.br

Affiliations:

1 Carlos Chagas Filho Biophysics Institute (IBCCF), Universidade Federal do Rio de Janeiro, Rio de Janeiro, Brazil

2 Department of Evolutionary Genetics, Leibniz Institute for Zoo and Wildlife Research, Berlin, Germany

3 Berlin Center for Genomics in Biodiversity Research, Berlin, Germany

4 Department of Science and Technological Innovation (DiSIT), Università del Piemonte Orientale Amedeo Avogadro, Vercelli-Novara-Alessandria, Italy

5 Wellcome Trust Sanger Institute, Wellcome Genome Campus, Hinxton CB10 1SA, UK

6 Centre of Immunobiology, Institute of Infection, Immunity & Inflammation, College of Medical, Veterinary and Life Sciences, University of Glasgow, Glasgow, UK.

7 Leopoldo de Meis Biomedical Biochemistry Institute (IBqM), Universidade Federal do Rio de Janeiro, Rio de Janeiro, Brazil

8 Bioinformatics Laboratory (LabInfo) of the National Laboratory for Scientific Computing, Petrópolis, Rio de Janeiro, Brazil

*Correspondence: marcela.uliano@gmail.com; mrebelo@biof.ufrj.br

ABSTRACT

Background: For more than 25 years, the golden mussel *Limnoperna fortunei* has aggressively invaded South American freshwaters, having travelled more than 5,000 km upstream across five countries. Along the way, the golden mussel has outcompeted native species and economically harmed aquaculture, hydroelectric powers, and ship transit. We have sequenced the complete genome of the golden mussel to understand the molecular basis of its invasiveness and search for ways to control it. **Findings:** We assembled the 1.6 Gb genome into 20548 scaffolds with an N50 length of 312 Kb using a hybrid and hierarchical assembly strategy from short and long DNA reads and transcriptomes. A total of 60717 coding genes were inferred from a customized transcriptome-trained AUGUSTUS run. We also compared predicted protein sets with those of complete molluscan genomes, revealing an exacerbation of protein-binding domains in *L. fortunei*. **Conclusions:** We built one of the best bivalve genome assemblies available using a cost-effective approach using Illumina pair-end, mate pair, and PacBio long reads. We expect that the continuous and careful annotation of *L. fortunei*'s genome will contribute to the investigation of bivalve genetics, evolution, and invasiveness, as well as to the development of biotechnological tools for aquatic pest control.

KEYWORDS: Amazon; binding domain; bivalves; genomics; TLR; transposon.

DATA DESCRIPTION

The golden mussel *Limnoperna fortunei* is an Asian bivalve that arrived in the southern part of South America about 25 years ago [1]. Research suggests that *L. fortunei* was introduced in South America through ballast water of ships coming from Hong Kong or Korea [2]. It was found for the first time in the estuary of the La Plata River in 1991 [1]. Since then, it has moved ~5,000 km, invading upstream continental waters and reaching northern parts of the continent [3] leaving behind a track of great economic impact and environmental degradation [4]. The latest infestation was reported in 2016 in the São Francisco River, one of the main rivers in the Northeast of Brazil, with a 2,700 km riverbed that provides water to more than 14 million people. At Paulo Afonso, one of the main hydroelectric power plants in the São Francisco River, maintenance due to clogging of pipelines and corrosion caused by the golden mussel is estimated to cost U$ 700,000 per year (*personal communication, Mizael Gusmã, Chief Maintenance Engineer for Centrais Hidrelétricas do São Francisco – CHESF).*

A recent review has shown that, before arriving in South America, *L. fortunei* was already an invader in China. Originally from the Pearl River Basin, the golden mussel has traveled 1,500 km into the Yang Tse and the Yellow River basins, being limited further north only by the extreme natural barriers of Northern China [5]. Today, *L. fortunei* is found in the Paraguaizinho River, located only 150 km from the Teles-Pires River that belongs to the Alto Tapajós River Basin and is the first to directly connect with the Amazon River Basin [6]. Due to its fast dispersion rates, it is very likely that *L. fortunei* will reach the Amazon River Basin in the near future.

The reason why some freshwater bivalves, such as *L. fortunei*, *Dreissena polymorpha*, and *Corbicula fluminea*, are aggressive invaders is not fully understood. These bivalves present characteristics such as (i) tolerance to a wide range of environmental variables, (ii) short life span, (iii) early sexual maturation, and (iv) high reproductive rates that allow them to reach densities as high as 150,000 ind.m^{-2} over a year [7, 8] that may explain the aggressive behavior. On the other hand, these traits are not exclusive to invasive freshwater bivalves and do not explain how they outcompete native species and disperse so widely.

To the best of our knowledge, there are no reports of successful strategies to control the expansion of mussel invasion in industrial facilities. Bivalves can sense chemicals in the water and close their valves as a defensive response [9], making them tolerant to a wide range of chemical substances, including strong oxidants like chlorine [10]. Microencapsulated chemicals have shown better results in controlling mussel populations in closed environments [10, 11] but it is unlikely they would work in the wild. Currently, there is no effective and efficient approach to control the invasion by *L. fortunei*.

The genome sequence is one of the most relevant and informative descriptions of species biology. The genetic substrate of invasive populations, upon which natural selection operates, can be of primary importance to understand and control a biological invader [12, 13].

We have partially funded the golden mussel genome sequencing through a pioneer crowdfunding initiative in Brazil (www.catarse.me/genoma). In this campaign, we could raise around USD$ 20,000.00 at the same time we promoted scientific education and awareness in Brazil.

Here we present the first complete genome dataset for the invasive bivalve *Limnoperna fortunei*, assembled from short and long DNA reads and using a hybrid and hierarchical assembly strategy. This high-quality reference genome represents a substantial resource for further studies of genetics and evolution of mussels, as well as for the development of new tools for plague control.

Genome sequencing in short Illumina and long PacBio reads

Limnoperna fortunei mussels were collected from the Jacui River, Porto Alegre, Rio Grande do Sul, Brazil (29°59′29.3″S 51°16′24.0″W). Voucher specimens were housed at the zoological collection (specimen number: 19643) of the Biology Institute at the Universidade Federal do Rio de Janeiro, Brazil. For the genome assembly, a total of 3 individuals were sampled for DNA extraction from gills and to produce the three types of DNA libraries used in this study. DNA was extracted using DNeasy Blood & Tissue Kit (Qiagen, Hilden, Germany) to prepare libraries for Illumina Nextera paired-end reads, with ~180bp and ~500bp of insert size, (ii) Illumina Nextera mate-pair reads with insert sizes from 3 to 15 Kb, and (iii) Pacific Biosciences long reads (**Table 1**). Illumina libraries were sequenced respectively in a HiScanSQ or HiSeq 1500 machine, and Pacific Biosciences reads were produced with the P4C6 chemistry and sequenced in 10 SMRT Cells. All Illumina reads were submitted to quality analysis with FastQC (FastQC, RRID:SCR_014583) followed by trimming with Trimmomatic (Trimmomatic, RRID:SCR_011848) [14]. Pacific Biosciences adaptor-free subreads sequences were used as input data for the genome assembly.

Table 1 - DNA reads produced for *L. fortunei* genome assembly

Library technology			Raw data		Trimmed Data*	
	**Reads insert size**	**Pairs**	**Number of reads**	**Number of bases**	**Number of reads**	**Number of bases**
**Illumina Nextera**	Paired end – 180 bp	R1	209542721	21060365702	209036571	21001101404
		R2	209542721	21049308698	209036571	20991650008
	Paired end – 500 bp	R1	153948902	15472966961	153482290	15423123500
		R2	153948902	15462883157	153482290	15414813589
	Mate pair 3-12 Kb	R1	178392944	18017687344	58157933	5822572152
		R2	178392944	18017687344	58157933	5811310412
**Pacific Biosciences**	P4C - 10/SMTRC	Subreads	1663730	11171487485		

*trimmomatic parameters for Illumina reads - ILLUMINACLIP:NexteraPE-PE.fa:2:30:10 SLIDINGWINDOW:4:2 LEADING:10 TRAILING:10 CROP:101 HEADCROP:0 MINLEN:80

For transcriptome sequencing, RNA was sampled from four tissues (gills, adductor muscle, digestive gland, and foot) of three different golden mussel specimens. RNA was extracted using NEXTflex Rapid Directional RNA-Seq Kit (Bioo Scientifics, TX, USA) and 12 barcodes from NEXTflex Barcodes compatible with Illumina NexSeq Machine. Resulting reads (**Supplementary Table S1**) were submitted to FastQC quality analysis (FastQC, RRID:SCR_014583) and trimmed with Trimmomatic (Trimmomatic, RRID:SCR_011848) [14] for all NEXTflex adaptors and barcodes. A total of 3 sets of *de novo* assembled transcriptomes

were generated using Trinity (Trinity, RRID:SCR_013048) **(Table 2**); one set for each specimen was a pool of the 4 tissue samples to avoid assembly bias due to intraspecific polymorphism [15]. All generated sequences are deposited in the SRA Archive under the following accession numbers: SRR5188384, SRR5195098, SRR5188200, SRR5195097, SRR5188315, and SRR5181514. Also this Whole Genome Shotgun project has been deposited in the DDBJ/ENA/GenBank under accession number NFUK00000000. The version described in this paper is version NFUK01000000. Genome files are available in the Gigascience database.

Table 2 - Trinity assembled transcripts used in the assembly and annotation of *L. fortunei* genome

Sample	Pooled tissues	Number of reads prior assembly	Number of Trinity Transcripts	Number of Trinity Genes	Average Contig Length	GC%
**Mussel 1**	Gills, mantle, digestive gland, foot	406589144	433197	303172	854	34
**Mussel 2**	Gills, mantle, digestive gland, foot	376577660	435054	298117	824	34
**Mussel 3**	Gills, mantle, digestive gland, foot	334316116	499392	351649	844	34

Genome assembly using a hybrid and hierarchical strategy

The Jellyfish software [16] was used to count and determine the distribution frequency of lengths 25 and 31 k-mers (**Figure 1**) for the Illumina DNA paired-end and mate-pair reads (**Table 1**). Genome size was estimated to be 1,6 Gb by using the 25 k-mer distribution plot as total k-mer number and then subtracting erroneous reads (starting k-mer counts from 12 times coverage), to further divide by the homozygous coverage-peak depth (45 times coverage), as performed by Li *et al.* (2010) [17]. A double-peak k-mer distribution was used as evidence of genome diploidy (**Figure 1**) and high heterozygosity. The rate of heterozygosity was estimated to be 2.07% and it was calculated as described by Vij *et al*. (2016) [18], using as input data the 25-kmer distribution plot for reads from one unique specimen.

Initially, we attempted to assemble the golden mussel genome using only short Illumina reads of different insert sizes (paired-end and mate-pairs, **Table 1**) using traditional *de novo* assembly software such as ALLPATHS [19], SOAPdenovo [20], and Masurca [21]. All these attempts resulted in very fragmented genome drafts, with an N50 no higher than 5 Kb and a total of 4 million scaffolds. To reduce fragmentation, we further sequenced additional long reads (10 PacBio SMTR Cells, **Table 1**) and performed a hybrid and hierarchical *de novo* assembly described below and depicted in **Figure 2**.

First, (i) trimmed paired-end and mate-pair DNA Illumina reads (**Table 1**) were assembled into contigs using the software Sparse Assembler [22] with parameters *LD 0 NodeCovTh 1 EdgeCovTh 0 k 31 g 15 PathCovTh 100 GS 1800000000*. Next, (ii) the resulting contigs were assembled into scaffolds using Pacific Biosciences long subreads data and the PacBio-correction-free assembly algorithm DBG2OLC [23] with parameters *LD1 0 k 17 KmerCovTh 10 MinOverlap 20 AdaptiveTh 0.01*. Finally, (iii) resulting scaffolds were submitted

to 6 iterative runs of the program L_RNA_Scaffolder [24] that uses exon-distance information from *de novo* assembled transcripts (**Table 2**) to fill gaps and connect scaffolds whenever appropriate. At the end, (iv) the final genome scaffolds were corrected for Illumina and Pacific Biosciences sequencing errors with the software PILON [25]: all DNA and RNA short Illumina reads were re-aligned back to the genome with BWA aligner (BWA , RRID:SCR_010910) [26] and resulting sam files were BAM-converted, sorted, and indexed with samtools package (SAMTOOLS, RRID:SCR_002105) [27]. Pilon [25] identifies INDELS and mismatches by coverage of reads and yields a final corrected genome draft. Pilon was run with parameters --*diploid –duplicates.*

The final genome was assembled in 20,548 scaffolds, with an N50 of 312 Kb and a total assembly length of 1.6 Gb (**Table 3**).

Table 3: **Assembly statistics for *Limnoperna fortunei*'s genome**

**Parameter**	**Value**
Estimated genome size by k-mer analysis	1.6 Gb
Total size of assembled genome	1.673 Gb
Number of scaffolds	20548
Number of contigs	61093
Scaffold N50	312 Kb
Maximum scaffold length	2.72 Mb
Percentage of genome in scaffolds > 50 Kb	82,55%
Masked percentage of total genome	33 %
Mapping percentage of Illumina reads back to scaffolds	91 %

The golden mussel genome presents 81% of all Benchmarking Universal Single Copy Orthologs (BUSCO version 3.3 analysis with Metazoa database) (BUSCO, RRID:SCR_015008) (**Table 4)** and, compared to the mollusk genomes currently available [28, 29, 30, 31, 32, 33, 34 35] it represents one of the best assemblies of molluscan genomes so far also in terms of scaffold N50 and contiguity **(Table 5)**.

One main challenges of assembling bivalve genomes lies in the high heterozygosity and amount of repetitive elements these organisms present: (i) the mussels *L. fortunei* and *Modiolus philippinarum* and the oyster *Crassostrea gigas* genomes were estimated to have heterozygosity rates of 2.07%, 2.02 % 1.95% respectively, which is substantially higher than other animal genomes [29], and (ii) repetitive elements correspond to at least 30% of the genomes of all studied bivalves so far (**Table 3**) [28, 29, 30, 31, 33, 34, 35]. Also, retroelements might be active in some species such as *L. fortunei* (refer to the retroelements-related section of this paper) and *C. gigas* [29], allowing genome rearrangements that may hinder for genome assembly. One exception seems to be the deep-sea mussel *B. platifrons* which has lower heterozygosity rates compared to other bivalves [31]. Sun *et al.,* (2017) [31] suggested it might be due to recurrent population bottlenecks happened after events of population extinction and recolonization in the extreme environment [31]. Nevertheless, most of the bivalve genome projects relying only on short Illumina reads are likely to present fragmented initial drafts [28, 30]. PacBio long reads allowed us to increase the N50 to 32 Kb and to reduce the number of scaffolds from millions to 61102, using the DBG2OLC [23] assembler. Finally, interactive runs of L_RNA_scaffolder [24] using the transcriptomes (**Table 2**) rendered the final result of N50 312 Kb in 20548 scaffolds. Thus, our assembly strategy of Illumina contigs, low coverage of PacBio reads, transcriptome

and Illumina re-mapping for final correction (**Figure 2**) represents an option for cost-efficient assembly of highly heterozygous genomes of nonmodel species such as bivalves.

Table 4: **Summary statistics of Benchmarking Universal Single-Copy Orthologs (BUSCO) analysis for *L. fortunei* genome run for Metazoans**

Categories	Number of Genes	Percentage (%)
Total BUSCO groups searched	978	--
Complete BUSCOs	801	81.9%
Complete and single-copy BUSCOs	769	78.62%
Complete and duplicated BUSCOs	32	3.27%
Fragmented BUSCOs	72	7.36%
Missing BUSCOs	105	10.73%

Table 5: Comparison of genome assembly statistics for molluscan genomes.

	Haliotis discus hannai	*Lottia gigantea*	*Aplysia californica*	*Ruditapes philippinarum*	*Patinopecten yessoensis*	*Crassostrea gigas*	*Pinctada fucata*	*Mytillus galloprovincialis*	*Bathymodiolus platifrons*	*Modiolus philippinarum*	***Limnoperna fortunei***
**Estimated genome size**	1.65Gb	359.5 Mb	1.8Gb	1.37 Gb	1.43 Gb	545 Mb	1.15 Gb	1.6 Gb	1.64Gb	2.38 Gb	**1.**6 Gb
**Number of scaffolds**	80,032	4,475	8,766	223,851	82,731	11,969	7997	1,746,447	65,664	74,575	**20,548**
**Total size of scaffolds**	1,865,475,499	359,512,207	715,791,924	2,561,070,351	987,685,017	558,601,156	915,721,316	1,599,211,957	1,659,280,971	2,629,649,654	**1,673,125,894**
**Longest scaffold**	2,207,537	9,386,848	1,784,514	572,939	7,498,238	1,964,558	5,897,787	67,529	2,790,175	715382	**2,720,304**
**Shortest scaffold**	854	1000	5001	500	200	100	1807	100	292	205	**558**
**Number of scaffolds > 1 K nt**	79,923 (99.9%)	4,471 (99.9%)	8,766 (100.0%)	138,771	16,004	5,788 (48.4%)	7997 (100%)	393,685 (22.5%)	38,704 (58.9%)	44,921 (60.2%)	**20,547 (100%)**
**Number of scaffolds > 1 M nt**	67 (0.1%)	98 (2.2%)	27 (0.3%)	0 (0.0%)	248 (0.3%)	60 (0.5%)	27 (0.3%)	0 (0.0%)	164 (0.2%)	0 (0%)	**95 (0.5%)**
**Mean scaffold size**	23,309	80,338	81,655	11,441	11,939	46,671	114,508	916	25,269	35,262	**81,425**
**Median scaffold size**	1,697	3,622	13,763	1,327	362	824	14,683	258	1,284	13,722	**22,134**
**N50 scaffold length**	200,099	1,870,055	264,327	48,447	803,631	401,319	345,846	2,651	343,373	100,161	**312,020**
**Sequencing coverage**	322 X	8.87 X	11 X	39.7 X	297 X	155 X	234 X	32 X	319 X	209.5 X	**60 X**
**Sequencing Technology**	Illumina + PacBio	Sanger	Sanger	Illumina	Illumina	Illumina	Illumina + BACs	Illumina	Illumina	Illumina	**Illumina + PacBio**

Around 10% of repetitive elements are transposons

Initial masking of *L. fortunei* genome was done using RepeatMasker program (RepeatMasker, RRID:SCR_012954) [36] with parameter -*species bivalves* and masked 3.4% of the total genome. This content was much lower than the masked portion of other molluscan genomes: 34% in *C. gigas* [29] and 36% in *M. galloprovincialis* [28], suggesting that the fast evolution of interspersed elements limits the use of repeat libraries from divergent taxa [37]. Thus, we generated a *de novo* repeat library for *L. fortunei* using the program RepeatModeler (RepeatModeler, RRID:SCR_015027) [38] and its integrated tools (RECON [39], TRF [40], and RepeatScout [41]). This *de novo* repeat library was the input to RepeatMasker together with the first masked genome draft of *L. fortunei*, and resulted in a final masking of 33.4% of the genome. Even though more than 90% of the repeats were not classified by RepeatMasker (**Supplementary Table S2**), 8.85% of the repeats were classified as LINEs, Class I transposable elements. In addition, large numbers of reverse-transcriptases (824 counts, Pfam RVT_1 PF00078), transposases (177 counts, Pfam HTH_Tnp_Tc3_2 PF01498), and integrases (501 counts, Pfam Retroviral integrase core domain PF00665) and other related elements were detected; over 98% of these had detectable transcripts.

More than 30,000 sequences identified by gene prediction and automated annotation.

To annotate the golden mussel genome, we sequenced a number of transcriptomes (**Table S1**), *de novo* assembled (**Table 2**) and aligned these transcriptomes to the genome scaffolds, and

created gene models with the PASA pipeline [36]. These models were used to train and run the *ab initio* gene predictor AUGUSTUS (Augustus: Gene Prediction, RRID:SCR_008417) [37] (**Supplementary Figure S1**). The complete gene models yielded by PASA [42] were BLASTed (e-value 1e-20) against the Uniprot database (UniProt, RRID:SCR_002380) and those with 90% or more of their sequences showing in the BLAST hit alignment were considered for further analysis. Next, all the necessary filters to run an AUGUSTUS [43] personalized training were performed: (i) only gene models with more than 3 exons were maintained, (ii) sequences with 90% or more overlap were withdrawn and only the longest sequences were retained, and (iii) only gene models free of repeat regions, as indicated by BLASTN similarity searches with *de novo* library of repeats, were maintained. These curated data yielded a final set of 1,721 gene models on which AUGUSTUS [35] was trained in order to predict genes in the genome using the default AUGUSTUS [43] parameters. Once the gene models were predicted, a final step was performed by using the PASA pipeline [42] once again in the *update* mode (parameters -c -A -g -t). This final step compared the 55,638 gene models predicted by AUGUSTUS [43] with the 40,780 initial transcript-based gene-structure models from PASA [42] to generate the final set of 60,717 gene models for *L. fortunei.* Of those, 58% had transcriptional evidence based on RNA Illumina reads (**Table S2**) re-mapping, rate that was expected since our RNA-Seq libraries were constructed only for 4 tissues of adult golden mussel specimens without any environmental stresses induction (**Table 2**). Therefore, these libraries lack transcripts for developmental stages, for some other cell types (i.e. hemocytes) and stress-inducible genes. Finally, 67% of the gene models were annotated by homology searches against Uniprot or NCBI NR (**Table 6**).

Table 6: Summary of gene annotation against various databases for *L. fortunei* whole genome-predicted genes

**Total number of genes**	60,717
**Total number of exons**	220,058
**Total number of proteins**	60,717
**Average protein size**	304 aa
**Number of protein BLAST hits* with Uniprot**	26,198
**Number of protein BLAST hits* with NR NCBI (no hits with Uniprot)**	14,810
**Number of protein HMMER hits* with Pfam.A**	24,513
**Number with proteins with KO assigned by KEGG**	8,387
**Number of proteins with BLAST hits* with EggNOG**	36,868

*all considered hits had a minimum e-value of 1e-05

Protein clustering indicates evolutionary proximity among mollusks species.

Gene family relationships were assigned using reciprocal best BLAST and OrthoMCL software (version 1.4) [44] between *L. fortunei* proteins and the total protein set predicted for nine other mollusks: the mussels *M. galloprovincialis*, *M. philippinarum* and *B. platifrons*, the clam *Ruditapes philippinarum*, the scallop *Patinopecten yessoensis*, the pacific oyster *C. gigas*,

the pearl oyster *Pinctada fucata* (genome version from Du *et al* [35]), and the gastropods *Lottia gigantea* and *Haliotis discus hannai* (see **Supplementary Table S3** for detailed information on the comparative data). **Figure 3A** presents orthologs relationships for five of the bivalves analyzed. A total of 6,337 orthologs groups are shared among the five bivalve species.

Of all the orthologous found for the total 10 species, 44 groups are composed of single-copy orthologs containing one representative protein sequence of each species. These sequences were used to reconstruct a phylogeny: the single-copy orthologs sequences were concatenated and aligned with CLUSTALW [45] with a resulting alignment of 30755 sites in length (**Figure 3B**). ProtTest 3.4.2 [46] was used to estimate the best fitting substitution model, which was VT [47]. With this alignment and model we reconstructed the phylogeny using PhyML [48] and 100 bootstrap repetition, the resulting tree is shown on **Figure 3B**.

Protein domain analysis shows expansion of binding domain in *L. fortunei.*

We performed a quantitative comparison of protein domains predicted from whole genome projects of 10 molluscan species. The complete protein sets of *M. galloprovincialis, M. philippinarum* and *B. platifrons*, *Ruditapes philippinarum*, *Patinopecten yessoensis, C. gigas*, *Pinctada fucata*, *Lottia gigantea* and *Haliotis discus hannai* (**Supplementary Table S3**) were submitted to domain annotation using HMMER against Pfam-A database (e-value 1e-05). Protein expansions in *L. fortunei* were rendered using the normalized Pfam count value (average) obtained from the other nine mollusks, according to a model based on the Poisson cumulative distribution. Bonferroni correction ($p \leq 0.05$) was applied for false discovery and absolute frequencies of Pfam-assigned-domains were initially normalized by the total count

number of Pfam-assigned-domains found in *L. fortunei* to compensate for discrepancies in genome size and annotation bias.

For *L. fortunei*, the annotation against Pfam.A classified 40127 domains in 24513 gene models of which 83 and 67 were respectively expanded or contracted in comparison with the other mollusks (**Supplementary Table S4** and **S5**; **Figure 4A**). The 83 overrepresented domains were further analyzed for functional enrichment using domain-centric Gene Ontology (**Figure 4B**). The analysis shows a prominent expansion of binding domains in *L. fortunei*, such as Thrombospondin (TSP_1), Collagen, Immunoglobulins (Ig, I-set,Izumo-Ig Ig_3), and Ankyrins (Ank_2, Ank_3, and Ank_4). These repeats have a variety of binding properties and are involved in cell-cell, protein-protein and receptor-ligand interactions driving evolutionary improvement of complex tissues and immune defense system in metazoans [49, 50, 51, 52, 53]. An evolutionary pressure towards the development of a diversificated innate immune system is also suggested by the high amount of Leucine Rich Repeats (LRR) and Toll/interleukin-1 receptor homology domains (TIR). Death, another over-represented PFAM, is also part of TLR signaling, being present in several docking proteins such as Myd88, Irak4 and Pelle [54]. Interestingly, BLAST analysis of *L. fortunei* gene models against Uniprot identified two types of Toll Like Receptors (TLRs) whose prototypical architecture of N-terminal extracellular leucine-rich repeat (LRR) motifs including either a single or multiple cysteine cluster domain, a C-terminal TIR domain spaced by a single transmembrane-spanning domain [55] could be correctly identified using the Simple Modular Architecture Research Tool (SMART) [56]. Indeed, we confirmed 141 sequences with similarity to single cysteine clusters TLRs (scc) typical of vertebrates, and 29 sequence hits with the multiple cysteine cluster TLRs (mcc) typical of *Drosophila* [55].

Phylogenetic analysis of all sequences (using PhyML [48], model JTT) (**Supplementary Figure S2**) shows evidence for TLRs clade separation in *L. fortunei*; the scc TLRs exhibit a higher degree of amino acid changes, higher molecular evolution, and diversification than the mcc TLRs. Overall, the expansion of these gene families might suggest an improved resistance to infections. It is, however, equally curious that other immune-related gene families such as Fribinogen_C and C1q seem to be contracted (**Supplementary Table S5**). This feature may depend on the evolutionary-driven, yet random, fate of the *L. fortunei* genome and consequence of different specific duplicate genes in other species. Also, other protein families involved in toxin metabolism, especially glutathione based processes and sulfotransferases are clearly contracted (**Table S5**).

Final considerations

Here we have described the first version of the golden mussel complete genome and its automated gene prediction that were funded through a crowdfunding initiative in Brazil. This genome contains valuable information for further evolutionary studies of bivalves and metazoa in general. Additionally, our team will further search for the presence of proteins of biotechnology interest such as the adhesive proteins produced by the foot gland that we have described elsewhere [57], or genes related to the reproductive system that have been shown to be very effective for invertebrate plague control [58]. The golden mussel genome and the predicted proteins are available for download in the Gigabase repository and the scientific community is welcome to further curate the gene predictions.

As the golden mussel advances towards the Amazon river basin, the information provided in this study may be used to help developing biotechnological strategies that may control the expansion of this organism in both industrial facilities and open environment.

Availability of supporting data

Limnoperna fortunei's genome and transcriptome data are available in the Sequence Read Archive (SRA) as BioProject PRJNA330677 and under the accession numbers **SRR5188384, SRR5195098, SRR518800, SRR5195097, SRR5188315, SRR5181514.** Also this Whole Genome Shotgun project has been deposited in the DDBJ/ENA/GenBank under accession number NFUK00000000. The version described in this paper is version NFUK01000000.

Additional files

Supplementary Table S1. RNA raw reads sequenced for 3 *L. fortunei* specimens, 4 tissues each.

Supplementary Table S2: RepeatMasker classification of repeats predicted in *L. fortunei* genome.

Supplementary Table S3: Details of the online availability of the data used for ortholog assignment and protein domain expansion analysis.

Supplementary Table S4: Expanded protein families in *L. fortunei* genome.

Supplementary Table S5: Contracted protein families in L. *fortunei* genome.

Supplementary Table S6: Fantasy names given to *L. fortunei* genes and proteins from the backers that have supported us through crowdfunding (www.catarse.me/genoma).

Supplementary Figure 1: Steps performed for the prediction and annotation of *L. fortunei* genome.

Supplementary Figure 2: Phylogenetic tree of Toll-like (TLRs) receptors found in *L. fortunei* genome.

List of Abbreviations

BUSCO: Benchmarking Universal Single-Copy Orthologs; SRA: Sequence Read Archive; KEGG: Kyoto Encyclopedia of Genes and Genomes.

Competing interests

The authors declare that they have no competing interests.

Authors' contribution

Conceived and designed the experiments: MR, MU, TO, CM, FD. Performed the experiments: MU, JA. Analyzed the data: MU, TO, CM, FD, FP, NC, IC, MR. Contributed reagents/materials/analysis tools: MR, FP, CM. Wrote the paper: MU, FD, MR. All authors read and approved the final manuscript.

Funding

This work was supported by the Brazilian Government agencies CAPES (PVE 71/2013), FAPERJ APQ1 (2014), and FAPERJ/DFG (39/2014). Also, this work was funded through crowdfunding with the support of 346 people (www.catarse.me/genoma).

Acknowledgements

We thank Susan Mbedi and Kirsten Richter from BeGenDiv for RNA-Seq library preparation and sequencing. We thank Dr. Loris Bennett for IT support while performing bioinformatics analysis.

We especially want to thank the 346 backers that supported the sequencing of the golden mussel through crowdfunding, in a 2013 campaign that raised U$ 20,000.00 (www.catarse.me/genoma). We decided to give fantasy names to the genes and proteins that we found in the genome, to thank the backers for their support. The name list is available in **Supplementary Table S6**.

Consent for publication

Does not apply.

Ethics approval

Limnoperna fortunei specimens used for DNA extraction and sequencing were collected in the Jacuí River (29°59′29.3″S 51°16′24.0″W), southern Brazil. This bivalve is an exotic species in Brazil and is not characterized as an endangered or protected species.

References

1. Pastorino G, Darrigran G, et al.,. Limnoperna fortunei (Dunker, 1857) (Mytilidae), nuevo bivalvo invasor em águas Del Rio de la Plata. Neotropica. 1993;39:101–2.
2. Darrigran G. Potential impact of filter-feeding invaders on temperate inland freshwater environments. *Biol Invasions* 2002; 4:145–156.

3. Uliano-Silva M, Fernandes F da C, Holanda IBB, Rebelo MF. Invasive species as a threat to biodiversity: The golden mussel *Limnoperna fortunei* approaching the Amazon River basin. In: Exploring Themes Aquat Toxicol Alodi, S, editor. Research Signpost; 2013.

4. Boltovskoy D, Correa N. Ecosystem impacts of the invasive bivalve Limnoperna fortunei (golden mussel) in South America. Hydrobiologia. 2015;746(1):81–95.

5. Xu M. Distribution and Spread of Limnoperna fortunei in China. In: Limnoperna fortunei Boltovskoy D, editor. Cham: Springer International Publishing; 2015 p. 313–20.

6. Oliveira M, Hamilton S, Jacobi C. Forecasting the expansion of the invasive golden mussel Limnoperna fortunei in Brazilian and North American rivers based on its occurrence in the Paraguay River and Pantanal wetland of Brazil. Aquat Invasions. 2010;5(1):59–73.

7. Karatayev AY, Boltovskoy D, Padilla DK, Burlakova LE. The invasive bivalves dreissena polymorpha and limnoperna fortunei: parallels, contrasts, potential spread and invasion impacts. J Shellfish Res. 2007 1;26(1):205–13.

8. Orensanz JM (Lobo), Schwindt E, Pastorino G, Bortolus A, Casas G, Darrigran G, et al. No Longer The Pristine Confines of the World Ocean: A Survey of Exotic Marine Species in the Southwestern Atlantic. Biol Invasions. 2002 1;4(1–2):115–43.

9. Claudi R and Mackie GL. Practical manual for zebra mussel monitoring and control. Lewis Publishers, Boca. Raton, Florida, 1994. p 227

10. Calazans SHC, Americo JA, Fernandes F da C, Aldridge DC, Rebelo M de F. Assessment of toxicity of dissolved and microencapsulated biocides for control of the Golden Mussel Limnoperna fortunei. Mar Environ Res. 2013 91:104–8.

11. Aldridge DC, Elliott P, Moggridge G.D. Microencapsulated biobullets for the control of biofouling zebra mussels. Environ. Sci. Technol. 2006 40:975-979.

12. Cox GW. Alien species and evolution: the evolutionary ecology of exotic plants, animals, microbes, and interacting native species. Washington: Island Press; 2004. 377 p.

13. Hall MR, Kocot KM, Baughman KW, Fernandez-Valverde SL, Gauthier MEA, Hatleberg WL, et al. The crown-of-thorns starfish genome as a guide for biocontrol of this coral reef pest. Nature. 2017 13;544(7649):231–4.

14. Bolger AM, Lohse M, Usadel B. Trimmomatic: A flexible trimmer for Illumina Sequence Data. Bioinformatics. 2014 1;170.

15. Trapnell C, Roberts A, Goff L, Pertea G, Kim D, Kelley DR, et al. Differential gene and transcript expression analysis of RNA-seq experiments with TopHat and Cufflinks. Nat Protoc. 2012 1;7(3):562–78.

16. Marçais G, Kingsford C. A Fast, Lock-free Approach for Efficient Parallel Counting of Occurrences of K-mers. Bioinformatics. 2011 Mar;27(6):764–770.

17. Li R, Fan W, Tian G, Zhu H, He L, Cai J, et al. The sequence and de novo assembly of the giant panda genome. Nature. 2010 Jan 21;463(7279):311–7.

18. Vij S, Kuhl H, Kuznetsova IS, Komissarov A, Yurchenko AA, Heusden PV, et al. Chromosomal-Level Assembly of the Asian Seabass Genome Using Long Sequence Reads and Multi-layered Scaffolding. PLOS Genet. 2016 Apr 15;12(4):e1005954.

19. Gnerre S, Maccallum I, Przybylski D, Ribeiro FJ, Burton JN, Walker BJ, et al. High-quality draft assemblies of mammalian genomes from massively parallel sequence data. Proc Natl Acad Sci U S A. 2011 25;108(4):1513–8.

20. Luo R, Liu B, Xie Y, Li Z, Huang W, Yuan J, et al. SOAPdenovo2: an empirically improved memory-efficient short-read de novo assembler. GigaScience. 2012;1:18.

21. Zimin AV, Marçais G, Puiu D, Roberts M, Salzberg SL, Yorke JA. The MaSuRCA genome assembler. Bioinformatics. 2013 1;29(21):2669–77.

22. Ye C, Ma Z, Cannon CH, Pop M, Yu DW. Exploiting sparseness in de novo genome assembly. BMC Bioinformatics. 2012;13(Suppl 6):S1.

23. Ye C, Hill CM, Wu S, Ruan J, Ma Z (Sam). DBG2OLC: Efficient Assembly of Large Genomes Using Long Erroneous Reads of the Third Generation Sequencing Technologies. Sci Rep. 2016 30;6:31900.

24. Xue W, Li J-T, Zhu Y-P, Hou G-Y, Kong X-F, Kuang Y-Y, et al. L_RNA_scaffolder: scaffolding genomes with transcripts. BMC Genomics. 2013;14:604.

25. Walker BJ, Abeel T, Shea T, Priest M, Abouelliel A, Sakthikumar S, et al. Pilon: An Integrated Tool for Comprehensive Microbial Variant Detection and Genome Assembly Improvement. PLOS ONE. 2014 19;9(11):e112963.

26. Li H, Durbin R. Fast and accurate short read alignment with Burrows–Wheeler transform. Bioinformatics. 2009 15;25(14):1754–60.

27. Li H, Handsaker B, Wysoker A, Fennell T, Ruan J, Homer N, et al. The Sequence Alignment/Map format and SAMtools. Bioinformatics. 2009 15;25(16):2078–9.

28. Murgarella M, Puiu D, Novoa B, Figueras A, Posada D, Canchaya C. A First Insight into the Genome of the Filter-Feeder Mussel Mytilus galloprovincialis. PLOS ONE. 2016 15;11(3):e0151561.

29. Zhang G, Fang X, Guo X, Li L, Luo R, Xu F, et al. The oyster genome reveals stress adaptation and complexity of shell formation. Nature. 4;490(7418):49–54.

30. Takeuchi T, Kawashima T, Koyanagi R, Gyoja F, Tanaka M, Ikuta T, et al. Draft genome of the pearl oyster Pinctada fucata: a platform for understanding bivalve biology. DNA Res Int J Rapid Publ Rep Genes Genomes. 2012 19(2):117–30.

31. Sun J, Zhang Y, Xu T, Zhang Y, Mu H, Zhang Y, et al. Adaptation to deep-sea chemosynthetic environments as revealed by mussel genomes. Nat Ecol Evol. 2017 Apr 3;1(5):0121

32. Nam B-H, Kwak W, Kim Y-O, Kim D-G, Kong HJ, Kim W-J, et al. Genome sequence of pacific abalone (Haliotis discus hannai): the first draft genome in family Haliotidae. GigaScience. 2017 May;6(5):1–8.

33. Wang S, Zhang J, Jiao W, Li J, Xun X, Sun Y, et al. Scallop genome provides insights into evolution of bilaterian karyotype and development. Nat Ecol Evol. 2017 Apr 3;1(5):0120.

34. Mun S, Kim Y-J, Markkandan K, Shin W, Oh S, Woo J, et al. The Whole-Genome and Transcriptome of the Manila Clam (Ruditapes philippinarum). Genome Biol Evol. 2017 Jun;9(6):1487–98

35. Du X, Fan G, Jiao Y, Zhang H, Guo X, Huang R, et al. The pearl oyster Pinctada fucata martensii genome and multi-omic analyses provide insights into biomineralization. GigaScience. 2017 Aug;6(8):1–12

36. Smit AF., Hubley R, Green PJ. RepeatMasker Open-3.0. 1996 2010.

37. Fu H, Dooner HK. Intraspecific violation of genetic colinearity and its implications in maize. Proc Natl Acad Sci U S A. 2002 9;99(14):9573–8.

38. Smith AFA, Hubley R. RepeatModeler Open-1.0. [Internet]. 2014. Available from: http://www.repeatmasker.org

39. Bao Z, Eddy SR. Automated De Novo Identification of Repeat Sequence Families in Sequenced Genomes. Genome Res. 2002 12(8):1269–76.

40. Benson G. Tandem repeats finder: a program to analyze DNA sequences. Nucleic Acids Res. 1999 1;27(2):573–80.

41. Price AL, Jones NC, Pevzner PA. De novo identification of repeat families in large genomes. Bioinformatics. 2005 1;21(Suppl 1):i351–8

42. Haas BJ, Delcher AL, Mount SM, Wortman JR, Jr RKS, Hannick LI, et al. Improving the Arabidopsis genome annotation using maximal transcript alignment assemblies. Nucleic Acids Res. 2003 1;31(19):5654–66.

43. Stanke M, Diekhans M, Baertsch R, Haussler D. Using native and syntenically mapped cDNA alignments to improve de novo gene finding. Bioinforma Oxf Engl. 2008 1;24(5):637–44.

44. Li L, Stoeckert CJ, Roos DS. OrthoMCL: Identification of Ortholog Groups for Eukaryotic Genomes. Genome Res. 2003 1;13(9):2178–89.

45. Thompson JD, Higgins DG, Gibson TJ. CLUSTAL W: improving the sensitivity of progressive multiple sequence alignment through sequence weighting, position-specific gap penalties and weight matrix choice. Nucleic Acids Res. 1994 11;22(22):4673–80.

46. Darriba D, Taboada GL, Doallo R, Posada D. ProtTest 3: fast selection of best-fit models of protein evolution. Bioinformatics, 2011 27:1164-1165.

47. Müller T, Vingron M. Modeling amino acid replacement. J. Comput Biol. 2000. 7:761-776.

48. Guindon S, Gascuel O. A simple, fast, and accurate algorithm to estimate large phylogenies by maximum likelihood. Syst Biol. 2003. 52: 696-704.

49. Björklund ÅK, Ekman D, Elofsson A. Expansion of Protein Domain Repeats. PLoS Comput Biol. 2006;2(8):e114.

50. Rennemeier C, Hammerschmidt S, Niemann S, Inamura S, Zähringer U, Kehrel BE. Thrombospondin-1 promotes cellular adherence of gram-positive pathogens via recognition of peptidoglycan. FASEB J Off Publ Fed Am Soc Exp Biol. 2007 21(12):3118–32.

51. Schmucker D, Chen B. Dscam and DSCAM: complex genes in simple animals, complex animals yet simple genes. Genes Dev. 2009 15;23(2):147–56.

52. Pancer Z, Amemiya CT, Ehrhardt GRA, Ceitlin J, Larry Gartland G, Cooper MD. Somatic diversification of variable lymphocyte receptors in the agnathan sea lamprey. Nature. 2004 Jul 8;430(6996):174–80.

53. Tucker RP. The thrombospondin type 1 repeat superfamily. Int J Biochem Cell Biol. 2004 36(6):969–74.

54. Park HH, Lo YC, Lin SC, Wang L, Yang, JK, Wu H. The death domain superfamily in intracellular signaling of apoptosis and inflammation. Annu. Rev. Immunol. 2007 25, 561-586.

55. Leulier F, Lemaitre B..Toll-like receptors—taking an evolutionary approach. Nature Reviews Genetics, 2008 9,3: 165-178.

56. Schultz J, Milpetz F, Bork P, Ponting CP. SMART, a simple modular architecture research tool: identification of signaling domains. Proc Natl Acad Sci U S A. 1998 26;95(11):5857–64

57. Uliano-Silva M, Americo JA, Brindeiro R, Dondero F, Prosdocimi F, Rebelo M de F. Gene discovery through transcriptome sequencing for the invasive mussel Limnoperna fortunei. PloS One. 2014;9(7):e10297.

58. Hammond A, Galizi R, Kyrou K, Simoni A, Siniscalchi C, Katsanos D, et al. A CRISPR-Cas9 gene drive system targeting female reproduction in the malaria mosquito vector Anopheles gambiae. Nat Biotechnol. 2015 7;34(1):78–83.

59.

Figure 1: K-mer distribution of *Limnoperna fortunei* Illumina DNA reads (Table 1).

Figure 2: **Hierarchical assembly strategy employed for the golden mussel genome assembly.** Trimmed Illumina reads were assembled to the level of contigs with Sparse Assembler algorithm **(Step 1)**. Then, Illumina contigs and PacBio reads were used to build scaffolds with DBG2OLC assembler, that anchors Illumina contigs to erroneous PacBio subreads, correcting them and building longer scaffolds **(Step 2)**, followed by transcriptome joining scaffolds using L_RNA_scaffolder **(Step 3)**. Final scaffolds were corrected by re-aligning all Illumina DNA and RNA-seq reads back to them and calling consensus with Pilon software **(Step 4)**. In bold is bioinformatics software used in each step. Red blocks indicate PacBio errors, which are represented by insertions and/or deletions found in approximately 12% of PacBio subreads.

Figure 3A: Gene family assigned with OrthoMCL for the total set of proteins predicted from five mussel genome projects. Outside the Venn diagram its represented the species name and below it is the number of proteins / number of clustered proteins / number of clusters. **B**: Phylogeny of the concatenated data set using 44 single-copy orthologs extracted from ten molluscan genomes. The VT model was estimated to be best fitting substitution model with ProtTest 3.4.2. We reconstructed the phylogeny using PhyML and 100 bootstrap repetition.

Figure 4: Gene family representation analysis in the *L. fortunei* genome. Panel A. PFAM hierarchical clustering, heatmap. Features were selected according to a model based on the Poisson cumulative distribution of each PFAM count in the golden mussel genome vs the normalized average values found in the other nine molluscan genomes (Bonferroni correction, $P \leq 0.05$). Transposable elements were included in the analysis. Colors depict the log2 ratio between PFAM counts found in each single genome and the corresponding mean value. The hierarchical clustering used the average dot product for data matrix and complete linkage for

branching. Legend: Lf, *L. fortunei*;. Bp, *Bathymodioulus platifrons*; Mg, *Mytilus galloprovincialis*; Mp, *Modioulus philippinarum*; Cg, *Crassostrea gigas*; Pf, *Pinctada fucata*; Py, *Patinopecten yessoensis*; Rp, *Ruditapes philippinarum*; Hd, *Haliotus discus hannai*; Lg, *Lottia gigantea* **Panel B. Gene ontology analysis of expanded gene families (PFAMs), semantic scatter plot.** Shown are cluster representatives after redundancy reduction in a two-dimensional space applying multidimensional scaling to a matrix of semantic similarities of GO term. Color indicates the GO enrichment level (legend in upper left-hand corner); size indicates the relative frequency of each term in the UNIPROT database (larger bubbles represent less specific processes).

 Click here to download Figure Figure1-revised.png

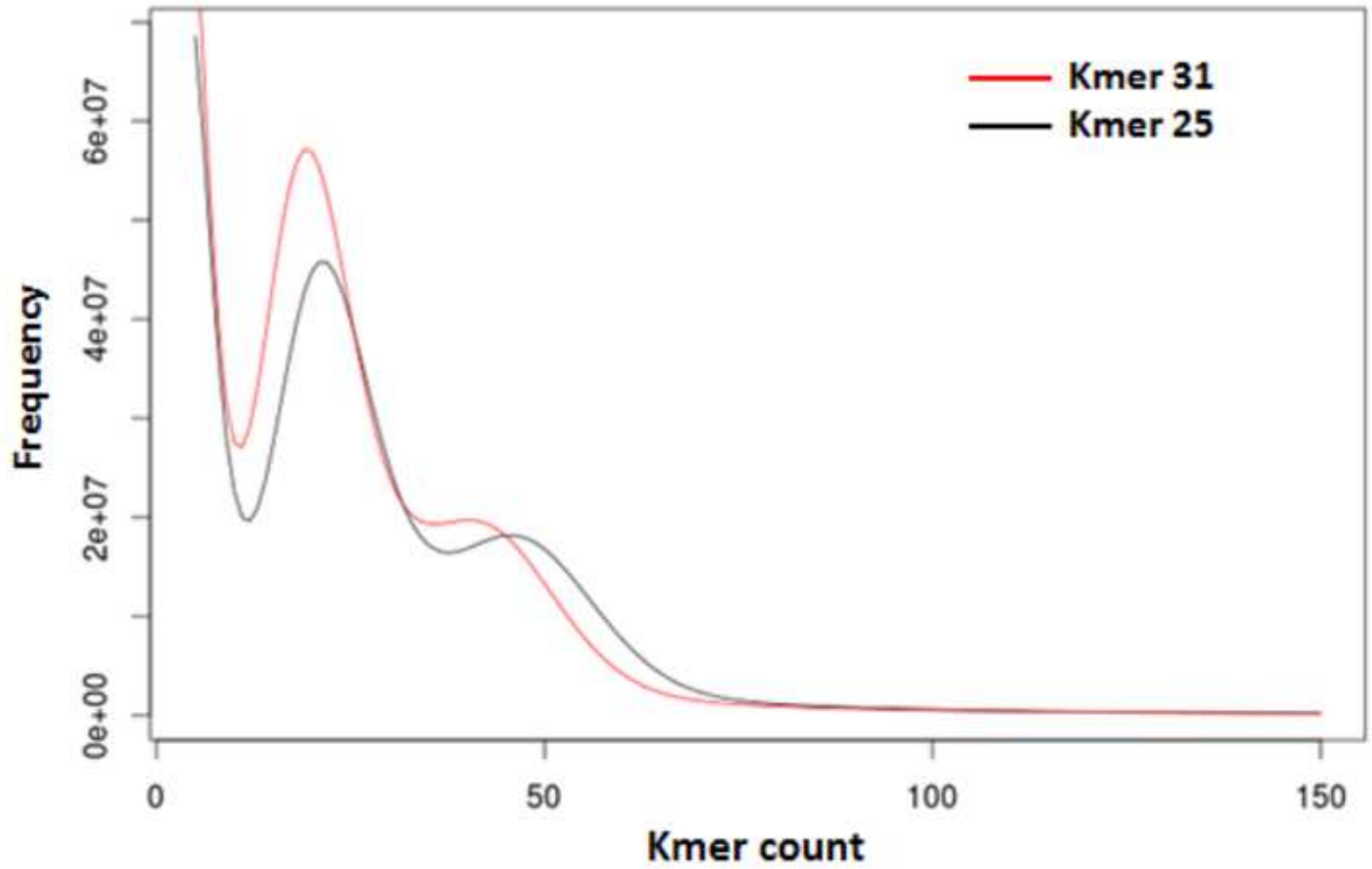

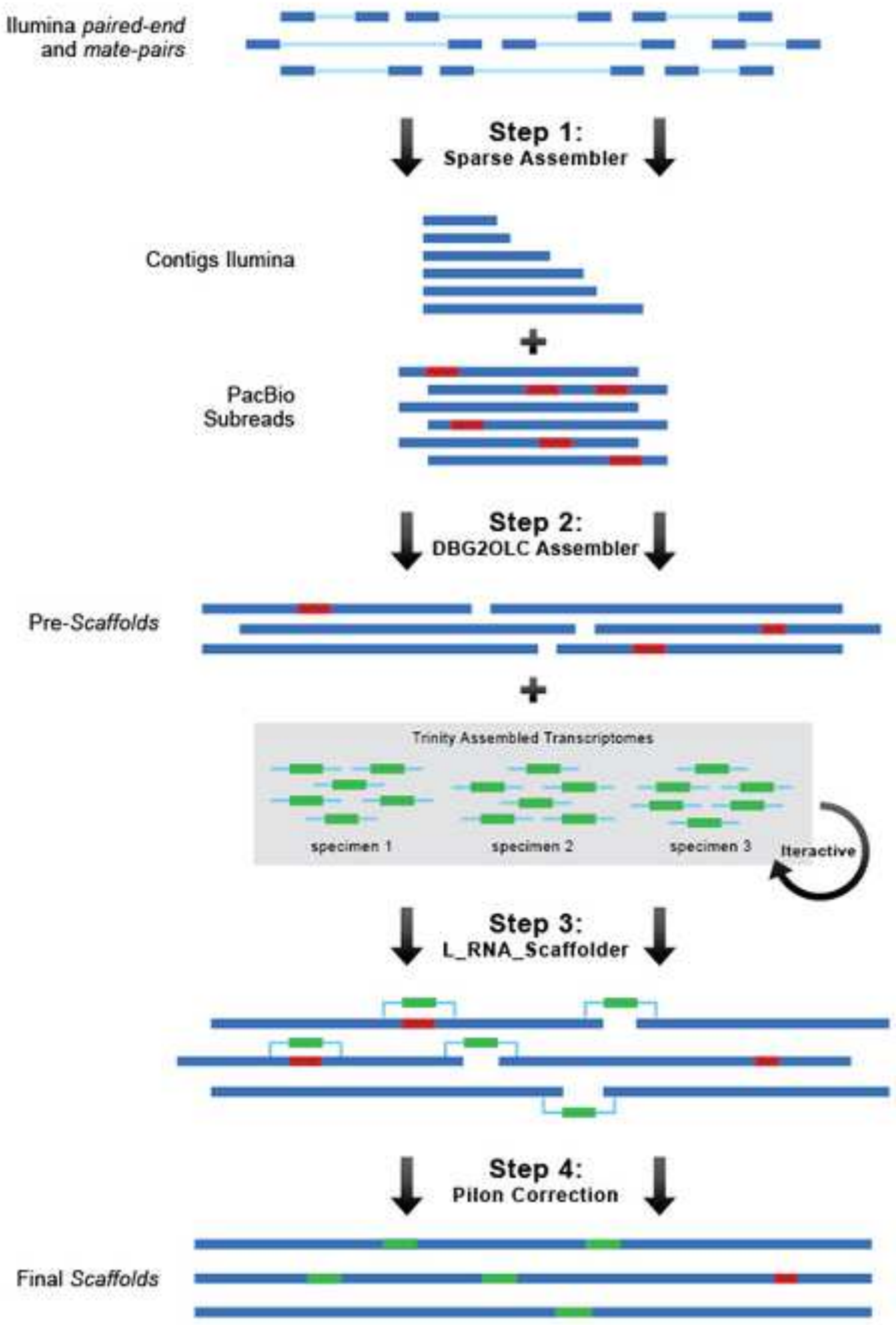
Ilumina *paired-end* and *mate-pairs*
Step 1:
Sparse Assembler
Contigs Ilumina
+
PacBio Subreads
Step 2:
DBG2OLC Assembler
Pre-*Scaffolds*
+
Trinity Assembled Transcriptomes
specimen 1
specimen 2
specimen 3
Iteractive
Step 3:
L_RNA_Scaffolder
Step 4:
Pilon Correction
Final *Scaffolds*

Figure3-revised

Click here to download Figure Figure3-revised.png

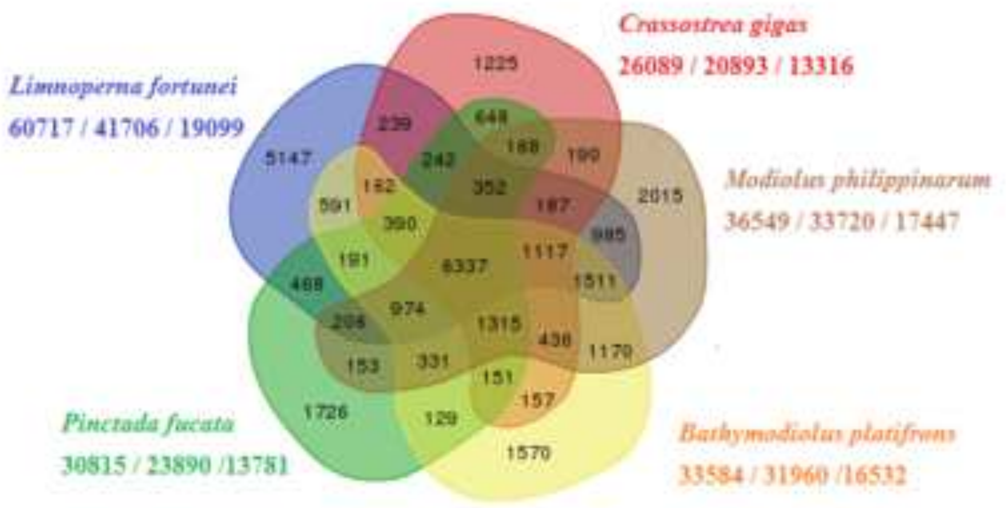

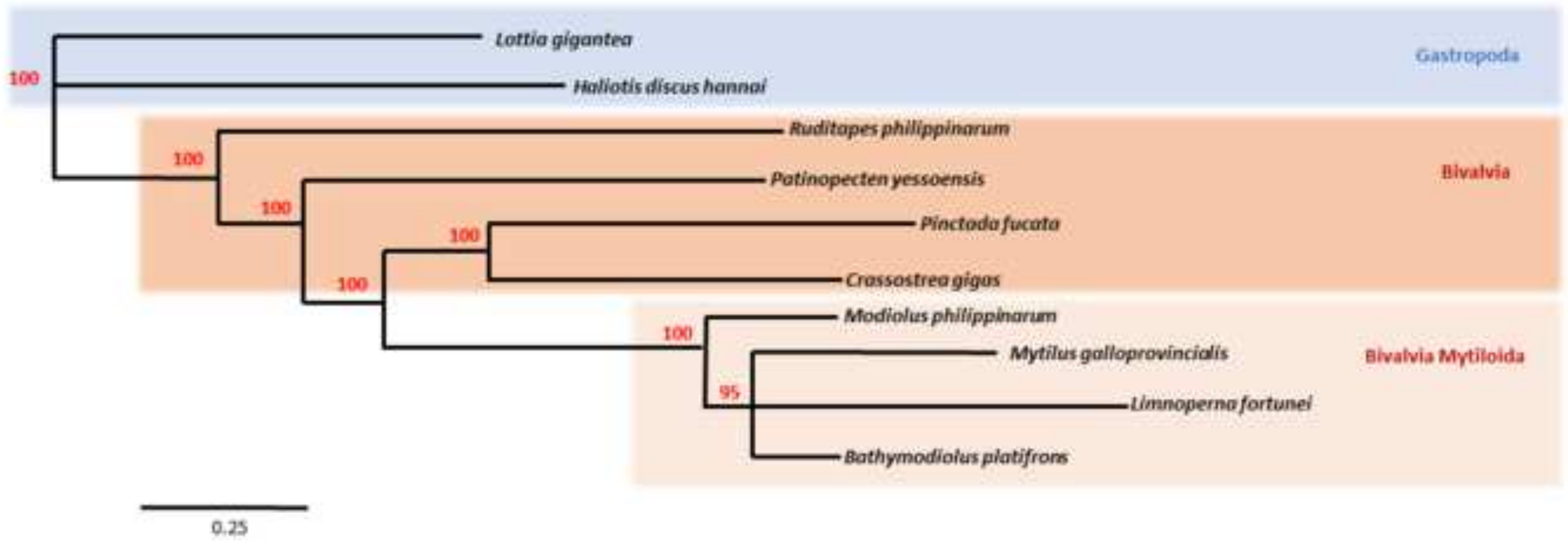

Figure4-revised

Click here to download Figure Figure 4- revised.pptx

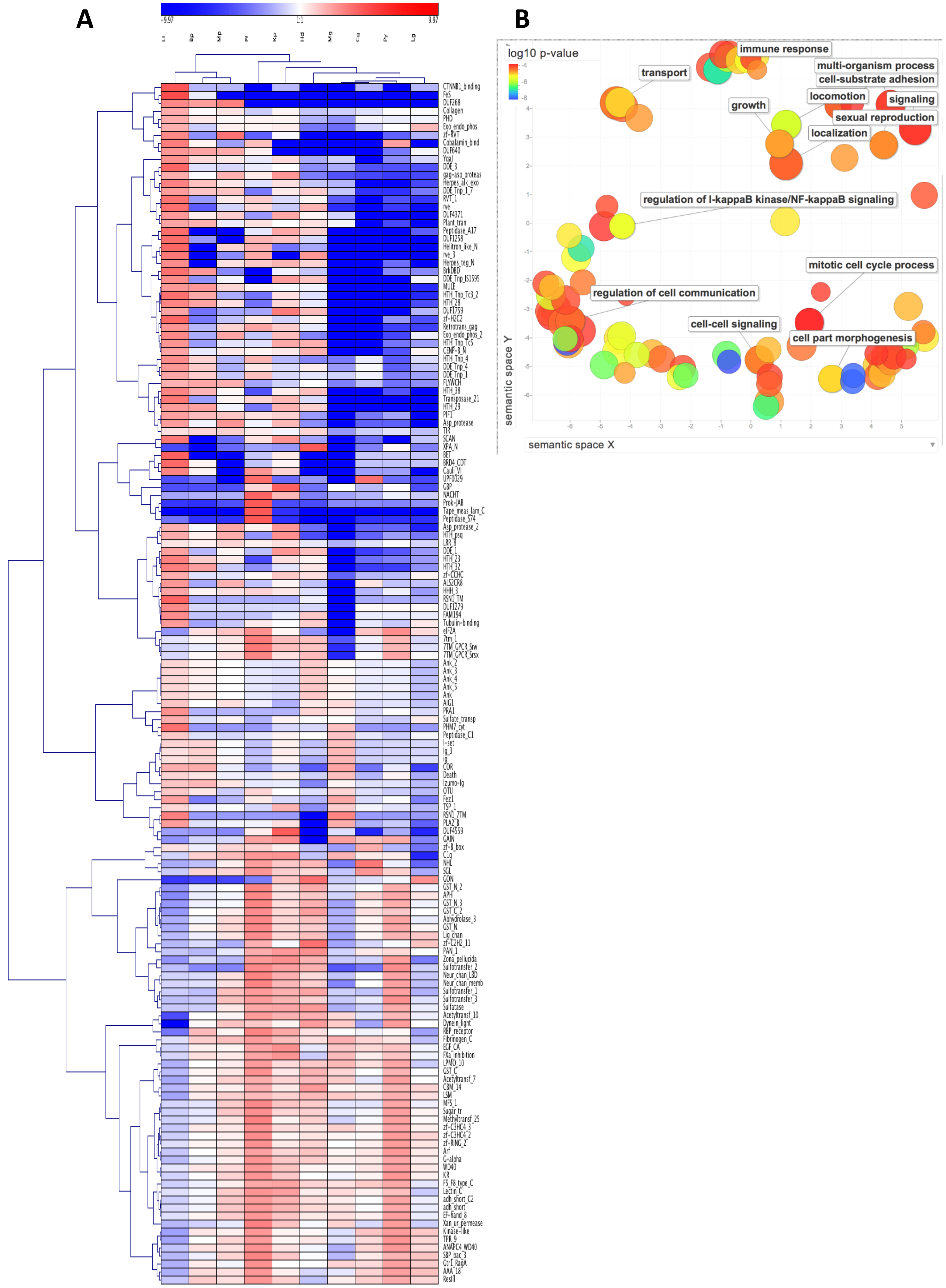

Table S1

Click here to access/download
Supplementary Material
TableS1.docx

Figure S1

Click here to access/download
Supplementary Material
Figure-S1.png

Table S2

Click here to access/download
Supplementary Material
TableS2.docx

Table S3

Click here to access/download
Supplementary Material
TableS3-revised.docx

Table S4

Click here to access/download
Supplementary Material
Table-S4-final.xlsx

Table S5

Click here to access/download
Supplementary Material
TableS5.xlsx

Figure S2

Click here to access/download
Supplementary Material
figureS2.pdf

Table S6

Click here to access/download
Supplementary Material
tableS6-.docx
