# Supplementary material for: A hybrid-hierarchical genome assembly strategy to sequence the invasive golden mussel, Limnoperna fortunei
Source: Gigascience. 2017 Dec 15;7(2):gix128. doi: 10.1093/gigascience/gix128 (PMC5836269; doi:10.1093/gigascience/gix128)
Supplement: Supplemental material [file gix128_supp.zip › TableS1.docx]

| Library technology - NEXTflexTM Rapid  Directional RNA-Seq Kit |  |  |  |  |  |  |
| --- | --- | --- | --- | --- | --- | --- |
| Reads | **Specimen** | **Tissue** | **Number of reads** | **Total number of reads per mussel** | **Number of reads** | **Number of bases** |
| R1 | Mussel 1 | gills | 30698278 |  | 30579413 | 4546689048 |
| R2 | Mussel 1 | gills | 30698278 |  | 30579413 | 4544749967 |
| R1 | Mussel 1 | mantle | 43247584 |  | 43083850 | 6429704391 |
| R2 | Mussel 1 | mantle | 43247584 |  | 43083850 | 6425857577 |
| R1 | Mussel 1 | digestive gland | 59371836 | 406589144 | 59150423 | 8859395587 |
| R2 | Mussel 1 | digestive gland | 59371836 |  | 59150423 | 8852588313 |
| R1 | Mussel 1 | foot | 69976874 |  | 69713300 | 10399401184 |
| R2 | Mussel 1 | foot | 69976874 |  | 69713300 | 10392761865 |
|  |  |  |  |  |  |  |
| R1 | Mussel 2 | gills | 37761566 |  | 37620655 | 5632300414 |
| R2 | Mussel 2 | gills | 37761566 |  | 37620655 | 5627830625 |
| R1 | Mussel 2 | mantle | 52359859 |  | 52165185 | 7785711260 |
| R2 | Mussel 2 | mantle | 52359859 | 376577660 | 52165185 | 7780769159 |
| R1 | Mussel 2 | digestive gland | 51477288 |  | 51279760 | 7643858942 |
| R2 | Mussel 2 | digestive gland | 51477288 |  | 51279760 | 7639425982 |
| R1 | Mussel 2 | foot | 46690117 |  | 46481387 | 6937080076 |
| R2 | Mussel 2 | foot | 46690117 |  | 46481387 | 6932038554 |
|  |  |  |  |  |  |  |
| R1 | Mussel 3 | gills | 42715593 |  | 42553887 | 6351524437 |
| R2 | Mussel 3 | gills | 42715593 |  | 42553887 | 6347642156 |
| R1 | Mussel 3 | mantle | 40727982 |  | 40576777 | 6052988849 |
| R2 | Mussel 3 | mantle | 40727982 | 334316116 | 40576777 | 6049207323 |
| R1 | Mussel 3 | digestive gland | 34672831 |  | 34529461 | 5157983507 |
| R2 | Mussel 3 | digestive gland | 34672831 |  | 34529461 | 5155094506 |
| R1 | Mussel 3 | foot | 49041652 |  | 48852467 | 7284483226 |
| R2 | Mussel 3 | foot | 49041652 |  | 48852467 | 7278546441 |

**Table S1**. RNA raw reads sequenced for 3 *L. fortunei* specimens, 4 tissues each
