# Supplementary material for: A hybrid-hierarchical genome assembly strategy to sequence the invasive golden mussel, Limnoperna fortunei
Source: Gigascience. 2017 Dec 15;7(2):gix128. doi: 10.1093/gigascience/gix128 (PMC5836269; doi:10.1093/gigascience/gix128)
Supplement: Supplemental material [file gix128_supp.zip › TableS2.docx]

| Total bases masked: 544,534,343 bp ( 32,55 %) |  |  |  |
| --- | --- | --- | --- |
|  | **Number of elements** | **Length occupied (bp)** | **Percentage of sequence (%)** |
| LINEs:  LINE2  L3/CR1 | 103,883  8,807   2,598 | 48,201,872  3,755,784  1,236,481 | 2.88  0.22  0.07 |
| LTR elements | 4,699 | 3,827,564 | 0.23 |
| DNA elements | 11,813 | 3,949,808 | 0.24 |
| Unclassified | 1,602,684 | 469,500,525 | 28.06 |
| Total interspersed repeats |  | 52,5479,769 | 31.41 |
| Simple repeats | 263,315 | 16,372,764 | 0.98 |
| Low complexity | 53,130 | 2,979,981 | 0.18 |

**Table S2**: Masking classification of the golden mussel *Limnoperna fortunei* genome performed by Repeat Masker.
