# Supplementary material for: A hybrid-hierarchical genome assembly strategy to sequence the invasive golden mussel, Limnoperna fortunei
Source: Gigascience. 2017 Dec 15;7(2):gix128. doi: 10.1093/gigascience/gix128 (PMC5836269; doi:10.1093/gigascience/gix128)
Supplement: Supplemental material [file gix128_supp.zip › TableS3-revised.docx]

**Supplementary Table S3**: Details of the online availability of the nine other mollusk genomes used for ortholog assignment and protein domain expansion analysis

| **Species** | **Protein Number** | **Genome size** | **Sequencing strategies** | **Online address** |
| --- | --- | --- | --- | --- |
| *Lottia gigantea* | 23340 | 359.5 Mb | Sanger | NCBI (BioProject PRJNA175706) |
| *Halotis discus hannai* | 29449 | 1.65 Gb | Illumina and Pacbio | GigaScience DB |
| *Crassostrea gigas* | 26089 | 545 Mb | Illumina and fosmids | www.esembl.org |
| *Pinctada fucata* | 30815 | 1.15 Gb | Illumina + BACs | *Giga*DB database |
| *Mytilus galloprovincialis* | 10891 | 1.6 Gb | Illumina | Requested to the corresponding author of the paper Murgarella *et al.,* (2016). |
| *Bathymoliolus platifrons* | 33584 | 1.64 Gb | Illumina | DRYAD Digital repository |
| *Modiolus philipparum* | 36549 | 2.38 Gb | Ilumina | DRYAD Digital repository |
| *Patinopecten yessoensis* | 26415 | 1.43 Gb | Illumina | PyBase: Patinopecten yessoensis Database |
| *Ruditapes philippinarum* | 108034 | 1.37 Gb | Illumina | Requested to the corresponding author of the paper Mun *et al.,* (2017). |
