# Supplementary material for: A hybrid-hierarchical genome assembly strategy to sequence the invasive golden mussel, Limnoperna fortunei
Source: Gigascience. 2017 Dec 15;7(2):gix128. doi: 10.1093/gigascience/gix128 (PMC5836269; doi:10.1093/gigascience/gix128)
Supplement: Supplemental material [file gix128_supp.zip › tableS6-.docx]

**Supplementary Information S4**

We are thankful to our crowdfunding backers for the funds provided to the golden mussel genome project (www.catarse.me/genoma). Here we present the fantasy names given to the genes and proteins of the golden mussel chosen by our backers:

Emilio Lanna - “Zeca” gene itr6_217_pil.g2715.t1 Lectin_C

Juliano de Mirando - “Izabela Damian dos Santos” gene HSP70

Rafael Oliveira Lopes - “Stella” stella itr6_3393_pi.g1519.t1 Actin

Eduardo Lam - “Charlie Chaplin” gene itr6_6314_pi.g523.t1 Ank_2

Ricardo Entz - “thebigheads” gene itr6_6314_pi.g523.t1 Ank_3

Gabriela Decker Sardinha - “Autonomista” gene itr6_3107_pi.g2181.t1 Ank_4

Marco Möller - “möllerina” gene itr6_2336_pi.g1026.t1 HSP70

André Dutra - “dedrutase” itr6_2408_pi.g1693.t1 Pkinase

Silvana Alodi - “allodina” itr6_5773_pi.g1093.t1 Phospholip_A2_1

Lucas Rech de Oliveira - “Caito” gene itr6_3504_pi.g2410.t1 F_actin_cap_B

Rodrigo Ligabeu Baun - “ligabuebraunin” gene itr6_4495_pi.g10.t1 GDI PF00996.16

Rodrigo Ligabeu Baun - “crichtonin“ gene itr6_490_pil.g2361.t1 HSP70

Roberto Heidi Utima - “Roberto Heidi Utima” gene itr6_329_pil.g72.t1 Lectin_C

Daniele Diniz Warken - “carlosew” gene itr6_531_pil.g2958.t1 Lectin_C

Orlando Hill - “Lilian Hill” gene itr6_4173_pi.g1818.t1 Lipase

Cecilia Fonseca Poggian - “Ceciliase” gene itr6_2116_pi.g2643.t1 Pkinase

Patrícia Sanae Sujii - “CienciaInformativa” gene itr6_4936_pi.g640.t1 zf-B_box

João Doria - “O Nicho Podcast” gene itr6_3030_pi.g1409.t1 HSP70

Davi Adorna - “Rosa Luxemburg” gene itr6_2336_pi.g1023.t1 HSP70

Fabiana Hecht - “SORIEDEM” gene itr6_3279_pi.g521.t1 Cadherin

Igor Drumond - “h2g2” gene itr6_16_pilo.g281.t1 HSP90

Anna Beatriz Robottom Ferreira - “Xuxuase” gene itr6_3863_pi.g124.t1 Pkinase

Thiago Augusto Pimenta Viana - “TAPVianase” gene itr6_2077_pi.g2107.t1.1.57f6923d Pkinase

Marcelo Simôes Resende - “Araguari Rezende” gene itr6_3081_pi.g1951.t1 Pkinase_Tyr

Mariana Magalhães Ferreira - "IRINEUSA" gene itr6_1966_pi.g589.t1 Actin

Jéssica de Aguiar França - "Ellen Tanus" gene itr6_4188_pi.g61.t1 Actin

Matheus Hobold Sovernigo - "HOBOLDSOVERNIGO" gene itr6_7439_pi.g48.t1 Ig_3

Fernando Feitosa - "FFeitosa" gene itr6_25_pilo.g423.t1 C1q

Marília Lima - "Lodettase" gene itr6_1072_pi.g1506.t1 ABC_ATPase

Richard Hemmi Valente - "Asimov" gene itr6_4936_pi.g640.t1 zf-B_box

Wendy Sauerbronn de Campos Valente - "Boris" gene itr6_3873_pi.g172.t1 zf-B_box

Diogo Costa - “Dncosta” gene itr6_13182_p.g16.t1 Cadherin_2

Marcelo Pinhero - "Alvicurina" gene itr6_5879_pi.g275.t1 Cadherin_pro

Marcelo Pinheiro - "Vibnerina" gene itr6_2651_pi.g929.t1 Cadherin_C

Jaqueline Dupre Moreira Ferraz - "tereclautriciajaquelucasmari" gene itr6_11591_p.g31.t1 Actin

Lucas Junior Teodoro - "LJTEODORO" gene itr6_433_pil.g1506.t1 Actin

Caio Bustani Andrade - “Bustani” gene itr6_216_pil.g2696.t1 Acyl_transf_1

Patricia Dupre Moreira Ferraz - “teca” gene itr6_3393_pi.g1519.t1 Actin

Rafael Mesquita L. Madureira - “Lourdes Mesquita” gene - itr6_777_pil.g1882.t1 zf-BED

Márcia Hoshina - “Hoshina” gene itr6_3002_pi.g1089.t1 Acyl_transf_1

Fernando Denardi Cibulski - “Cibulskiase” gene itr6_2506_pi.g2822.t1 Acyl_transf_1

Marcos Kimura - “BiLuHK” gene itr6_4936_pi.g640.t1 zf-B_box

Caroline Santesso - “iluminase” gene itr6_2531_pi.g3128.t1 Actin

Marcos Tinoco - “Tinoco” gene itr6_5047_pi.g1171.t1 Actin

Alessandra Lehmen - “aleh1 Lehmen” gene itr6_3873_pi.g172.t1 zf-B_box

Manoela Gomes Baptista - “MGBR” gene itr6_11_pilo.g247.t1 zf-C2H2

Débora Andrade - “TEREDEBYASE” gene itr6_4188_pi.g70.t1 Actin

Diogo Biazus - “Biazus” gene itr6_750_pil.g1581.t1 Acyl_transf_1

Gracielle Vendrúsculo Braga - “Vendruscolase” gene itr6_6341_pi.g598.t1 Acyl_transf_1

André Sá - “Filo-Sá” gene itr6_13685_p.g116.t1

Sabrina da Silva - “SKoifman” gene itr6_136_pil.g1741.t1 zf-C3HC4

Gutembergue Carvalho- “Jampruca” gene itr6_4188_pi.g65.t1 Actin

Fabiano Marques - “Marquesfab” gene itr6_5433_pi.g987.t1 zf-CCHC

André Luiz Vasconcellos de Araújo - “Mexilhão feio” itr6_4515_pi.g108.t1 Vasa

Ayla Sant'Ana da Silva - “Yuki” gene itr6_4014_pi.g1026.t1 Actin

Juliana Zucco - “zuccina” gene itr6_4188_pi.g60.t1 Actin

William Jeferson - “foratemerase” gene itr6_4188_pi.g63.t1 Actin

Evelise Dambros da Luz - “Eveliase” gene itr6_391_pil.g982.t1 Actin

Marianne Kreusch - “Marcheto-Kreusch” gene itr6_4188_pi.g67.t1 Actin

Rafael Trevisan - “LabDef” gene itr6_1771_pi.g1993.t1 GSHPx PF002

Henrique Lemos Jucá - “jucalase” gene itr6_2431_pi.g1931.t1 Helicase_C

Cynthia Cardoso - “chesterase” gene itr6_4515_pi.g108.t1 Helicase_C

Vinicius Fernandes dos Santos -”cabessa” gene itr6_5034_pi.g1088.t1 Tubulin

Sergio Silvestre - “SantosSilvestre” gene itr6_8349_pi.g7.t1 Helicase_C

Carlos Eduardo Lessa Pissuto - “KAROLLOS” gene itr6_6341_pi.g596.t1 Acyl_transf_1

Rodrigo Augusto da Silva - “Rods_77” gene itr6_2453_pi.g2130.t1 zf-CCHH

Juan Pedro Alves Lopes - “MATLopes” gene itr6_20277_p.g18.t1 zf-HYPF

Karina Silveira - “Karinina AS” gene itr6_3744_pi.g1657.t1 Tubulin

Karina Silveria - “SaleteAndrade” gene itr6_1174_pi.g2699.t1 zf-LITAF-like

Karina Silveria - “OsmarSilveira” itr6_136_pil.g1741.t1 zf-RING_UBOX

Giovana Girardi - “Girardase” gene itr6_1341_pi.g723.t1 Helicase_C

David Majerowicz - “Marfim” gene itr6_1407_pi.g1554.t1 Helicase_C

André Gomes Julião - “andrejuliao” gene itr6_1932_pi.g56.t1 Actin

Rodrigo de Oliveira Martins - “ROMBarnacleBoy” gene itr6_1320_pi.g423.t1 zf-RVT

Jackson Reis - “rafael.akira.i.f.reis” gene itr6_165_pil.g2044.t1 Tubulin

Jackson Reis - “Jackson.reis.f.s” gene itr6_12053_p.g97.t1 COesterase

Thiago Fialho - “Eliane Little” itr6_6314_pi.g523.t1 Ank_2

João Batista de Melo - “DANTEPEDeM” gene itr6_449_pil.g1726.t1 Helicase_C

Adriana de Lima Barbosa - "A.cohen" gene itr6_222_pil.g2782.t1 Tubulin

Fernando Ariel Genta - “Genta” gene itr6_3892_pi.g320.t1 Ank_2

Débora Lanznaster - “Edit Bruns” gene itr6_6314_pi.g523.t1 Ank_3

Mariana Souza da Silveira - “Duds” gene itr6_3107_pi.g2181.t1 Ank_4

Fabricio Docema de Oliveira - “Andréia de Oliveira Giannasi” gene itr6_5112_pi.g1392.t1 ATP-synt_E_2

Marcos Schettini - “Schettinina” gene itr6_165_pil.g2047.t1 Tubulin

Milena M. Schettini - "Milenar" gene itr6_2509_pi.g2892.t1 Lectin_C

Andre Campos Rodovalho - “verdade libertadora” gene itr6_3279_pi.g521.t1 Cadherin

Adriano Monteiro Castro - “Frida” itr6_1691_pi.g951.t1 Atrophin-1

Mariano Rodrigues Aloi - “maloizinho” gene itr6_222_pil.g2785.t1 Tubulin

Vinicius de Medeiros Alves - “Pequi” gene itr6_5034_pi.g1085.t1 Tubulin

Guilherme Machado Nunes - “proteína brasileira” itr6_3438_pi.g1869.t1 Tubulin

Juliano Bortolozzo Solanho - “Juaum Du Shops” itr6_3830_pi.g2323.t1 Tubulin

Pedro Camilo Maistro - “Maistro” gene itr6_1653_pi.g530.t1 Helicase_C

Marcus Leopoldino - “Aline de Souza Porto Leopoldino” gene itr6_5203_pi.g198.t1 C1q

Carolina Goulart - “hugo14” gene itr6_2297_pi.g572.t1 C1q

Gabriela Pimenta dos Reis - “P3004” gene itr6_1932_pi.g57.t1 Actin

Júlia Back Comandolli - “Catarina” gene itr6_165_pil.g2047.t1 Tubulin

Josafa Diniz Araujo Filho - “Josafa&Deisy” gene itr6_4042_pi.g1134.t1 ASC

Francis Graeff de Oliveira - “Fragui” gene itr6_165_pil.g2045.t1 Tubulin

Ricardo Alchini - “Alchini” gene itr6_19657_p.g45.t1 C2-set_2

Érico Gaiger Marshall - “erico” gene itr6_2108_pi.g2442.t1 AT_hook

Flávia Rachel Moreira Lamarão - “Flávia Lamarão” gene itr6_3369_pi.g1316.t1 Baculo_8kDa

Bruno Bottino Ferreira - “Marie Curie” gene itr6_4385_pi.g1272.t1 Actin

Gabriel Pereira Corrêa - “DANTE SRN” gene itr6_1653_pi.g530.t1 Helicase_C

Maria Gonzalez Rey - “Reynase” gene itr6_99_pilo.g1385.t1 Acyl_transf_1

Gustavo de Freitas - “Nicolas Gottschald de Freitas” gene itr6_4995_pi.g933.t1 Tubulin

Felipe Caruso - “Giordano Bruno” itr6_5273_pi.g429.t1 Actin

Vinicius Bassanese - “Valverina” gene itr6_433_pil.g1506.t1 Actin

Gustavo Seichi Inouye Shintate - “Shintatease” gene itr6_816_pil.g2496.t1 Acyl_transf_1

Aluisio Lucio da Rocha – “AJAKL MOREIRA ROCHA” gene itr6_1381_pi.g1250.t1 BAF

Marcio Rodrigues Paiva – “GaelRP” gene itr6_2624_pi.g531.t1 CABIT

Kelly Ribas Lobato - “OLB61015” gene itr6_364_pil.g614.t1 Cadherin

Aristides Neto – “Aristides Neto” gene itr6_1521_pi.g3025.t1 7TM_GPCR_Srw

Gabriela Pacheco Correa – “MARILUTP” gene itr6_5130_pi.g1452.t1 ANF_receptor

Bianca Lucchesi Targhetta – “Cientistas Feministas” gene itr6_4957_pi.g740.t1 Helicase_C

Yanna Koloniari Barbosa – “Yanna Koloniari” gene itr6_6314_pi.g523.t1 Ank_2

Renato Pinheiro Freme Lopes Lucindo – “Pupilu” gene itr6_2891_pi.g29.t1 Helicase_C

André E.O.P. Lico – “BRL_Mito” gene itr6_1691_pi.g951.t1 Atrophin-1

Eleonora Kurtenbach – “Olhos verdes” gene itr6_502_pil.g2473.t1 Helicase_C

Samuel Gales Guimarães – “Sanini Ayli-Lunia” gene itr6_4042_pi.g1134.t1 ASC

Marcela Davanso – “Cabeçase” gene itr6_15_pilo.g273.t1 Helicase_C

Márcio Pires Antonio – “Antônio” gene itr6_3892_pi.g320.t1 Ank_2

Diego Reeberg – “Catarse” gene itr6_3107_pi.g2181.t1 Ank_4

Diego Reeberg – “KatianiMartins” gene itr6_4252_pi.g398.t1 COesterase

Marcio Mazza – “Larissa” gene itr6_1483_pi.g2572.t1 CARD

Nicolas Iensen – “Nicolas Iensen” gene itr6_2531_pi.g3133.t1 Actin

Pedro Hutsch Balboni – “Pedro Hutsch Balboni” gene itr6_4255_pi.g438.t1 Tubulin

Igor Alves – “Rechetnicoase” gene itr6_2506_pi.g2820.t1 Acyl_transf_1

Daniele Botaro – “RafaBotarase” gene itr6_5047_pi.g1165.t1 Actin

Marcela Vargas dos Santos - “vargaslite” gene itr6_4188_pi.g58.t1 Actin

Mariana Martinhago - “Martinhaguina” gene itr6_774_pil.g1864.t1 CUB

Mariana Wagner da Rocha - “Bem” gene itr6_2918_pi.g255.t1 Defensin_2

Raquel Stoltz Back - “Back” gene itr6_1777_pi.g2105.t1 CARD

Maysa Ito - “swt8478” gene itr6_3830_pi.g2322.t1 Tubulin

Cássio Batista Marcon - “Tarobain” gene itr6_3015_pi.g1241.t1 Tubulin

Alexandre Fernandes - “A.Fernandes” galactose metabolism

Olavo Bohrer Amaral - “espelgenes” gene tr6_2974_pi.g796.t1 Death

Anne Leite - “ALFRL” gene tr6_1395_pi.g1387.t1 DAZAP2

Felipe Guimarâes Marques - Metabolic Pathway “Metabarques”. Names: ‘Fagner’, ‘Peed’, ‘Uomond’, ‘Gertrude’, ‘Animes’, ‘Cleide’, ‘Bognus’, ‘Jubblews’, ‘Entropinase’ and ‘Garori’.

Tomio Makihara - Metabolic Pathway “Protein Export”. Names: ‘T-TOMIO’, ‘T-SUELI’ ’T-CAROL’, ‘T-MARIANA’, ‘T-GABRIEL’ ‘T-FELIPE’, ‘T-ROMEO’, ‘T-MATEO’, ‘T-SERGIO’, ‘T-MARCELA’ and ‘T-MAURO’

Adalberto R. Vieyra – Metabolic Pathway “Vieyra“ Penthose Phosphate

Paulo Bandeira de Carvalho - “Bandeirase” gene itr6_5245_pi.g338.t1 Helicase_C

Júlia Crespo Viegas - “Viegano” gene itr6_754_pil.g1660.t1 Acyl_transf_3

Elis Amaral Rosa - “Rosalind Franklin” gene itr6_2974_pi.g796.t1 Death

Tatiana Alves Américo - “amerinda” gene itr6_13486_p.g3.t1 Actin

Túlio Baars – ‘ednina’ gene itr6_4188_pi.g64.t1 Actin

Cássia Rodrigues – ‘julianina’ gene itr6_1167_pi.g2579.t1 Tubulin

Emmnanuel Moura – Metabolic Pathway ‘Glutathione Metabolism’. Names: ‘Fidalgo’, ‘Furacão’, ‘Willow’, ‘Thanatos’, ‘Anubis’, ‘Ratak’, ‘Banshee’, ‘Cheshire’,  ‘Brenda’, ‘Gauss’, ‘Teddie’ and ‘Épée’.

Alexandre Fernandes e Souza – Metabolic Pathway “Fernandes e Souza” Galactose

Ricardo Antônio Rubens Prado Schneider – Metabolic Pathway “Schneider” Cytochrome P450

Turini Alberto – Metabolic Pathway “Turini” Seleno Compounds

Marzia Terrizzano – Metabolic Pathway “Terizzano” Folate

Red Bull Amaphiko – Metabolic Pathway “Amaphiko” Piruvate

Gustavo do Amaral Martins – Metabolic Pathway “Martins” RNA-polimerase

Fábio Gouveia – ‘Biel’ gene itr6_4081_pi.g1281.t1  Acyl_transf_3

Fábio Gouveia - ‘anagoya’ gene itr6_3142_pi.g2437.t1  Helicase_C

Fábio Gouveia - ‘bibipeg’  gene itr6_4725_pi.g1226.t1  Death

Gustavo Monnerat – ‘fundão” gene itr6_3771_pi.g1860.t1  Helicase_C

Leonardo Eloi – ‘eloína’ gene itr6_4081_pi.g1282.t1  Acyl_transf_3

Radical Livre – ‘LulaBonitase’ gene itr6_3458_pi.g2063.t1  Acyl_transf_3

**We are thankful to all our 346 backers in this project**. A few of them have already named genes in the mitochondrial genome (Uliano-Silva *et al.*, 2016).

We also thank:

Daniel Andrade Moreira, Camila M. Borges, Ingrid E. Zandomeneco, Vinicius F. dos Santos, Uilian Americo, Helder da Rocha, Heitor Dias Murbach, Juliana C. Fernandes, Caio W. P. Ferreira, Samuel W. L. Nery, Renata Minerbo, Terravixta Estudio, Guilherme A. Palma, Aurélio C. M. Moura, Danilo N. da Silva, Eduardo K. Amaraes, Rodrigo Zanatta, Júlia A. Kist, Andrea R. Balle, Julia Freitas, Rafael Chaves, Ricardo de Souza dos Reis, Sabrina Alves, Tiago F. A. A. Leite, Rafael dos Santos, Ana Carolina P. da Costa, Larissa M. Feijó, Leticia A. Z. Costa, Luiz Sauerbronn, Guilherme L. Tosi, Aline M. Ghilardi, Mayra S. Soares, Luiz Felipe S. dos Santos, Humberto P. Figueira, Gabriela B. Ramos, Lucas M. C. Brouck, Marcos C. Maleson, Laura S. Daros, Gabriel M. Vespucci, Robson R. Sebastião, Valeria Kneipp Sena, Luciano Caletti, Vivian Kahl, Rodolfo H. de Saboia, Leandro L. Rebelo, Otavio A, Cardoso, Jean Remy Davee Guimarães, Thum Thompson, Thiago E. Parente, André H. C. Silva, Ana Paula B. Moreira, Eduardo Sampaio, Janaína B. Resende, Felipe A. do Nascimento e Silva, Kleber L. de Carvalho, Alexandre M. Hartke, Lucas A. de Abreu, Victor Hugo Barbosa, Diego Remus, Frederico Q. do Amaral, Júlia Lima, Rosane B. de Oliveira, Rafael S. Ozaki, Magno B. C. Branco, Tiago O. Rinaldi, Andrea F. Coutinho, André R. da Silva, Eliane S. Figueiredo, Eduardo Elael, Rafael F. Sampaio, Guacyra do C. Pereira, Henrique B. Gouveia, Glauber D. Ramos, Denis D. Maua, Vitor R. S. Cruz, Rafael M. Meyer, William Krein, Luiza A. L. Meireles, Daniel A. P. Martins, Rafael Dourado, Daniela S. M. Peres, Francisney P. do Nascimento, Diego P. Sérgio, Maurício H. de Mello, Jonatha C. B. das Neves, Heloisa A. Molleri, Tiago C. Alexandre, Anna L. F. Schubert, Christian R. Reis, Claudia A. M Russo, Débora T. A. da Silav, Flávia A. Ventura, Jeamnylle Nilin, Silvio Kozasa, Rogério M. Pastore, Daniela S. M. Peres, Diego P. Adorna, Marcos E. Maes, Igor Rechetnicow.
