# Supplementary figures and images for: A hybrid-hierarchical genome assembly strategy to sequence the invasive golden mussel, Limnoperna fortunei
Source: Gigascience. 2017 Dec 15;7(2):gix128. doi: 10.1093/gigascience/gix128 (PMC5836269; doi:10.1093/gigascience/gix128)

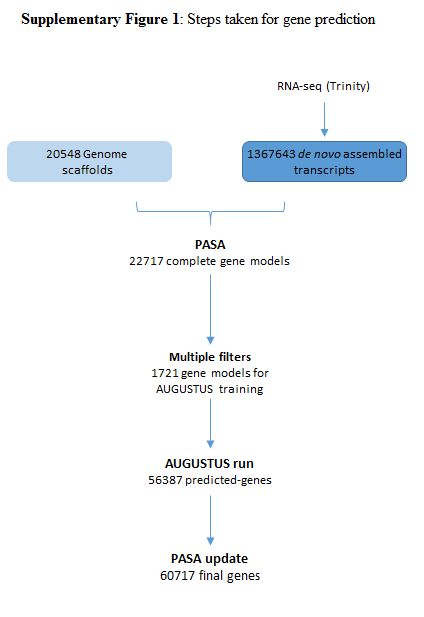

Supplement: Supplemental material [file gix128_supp.zip › Figure-S1.png]

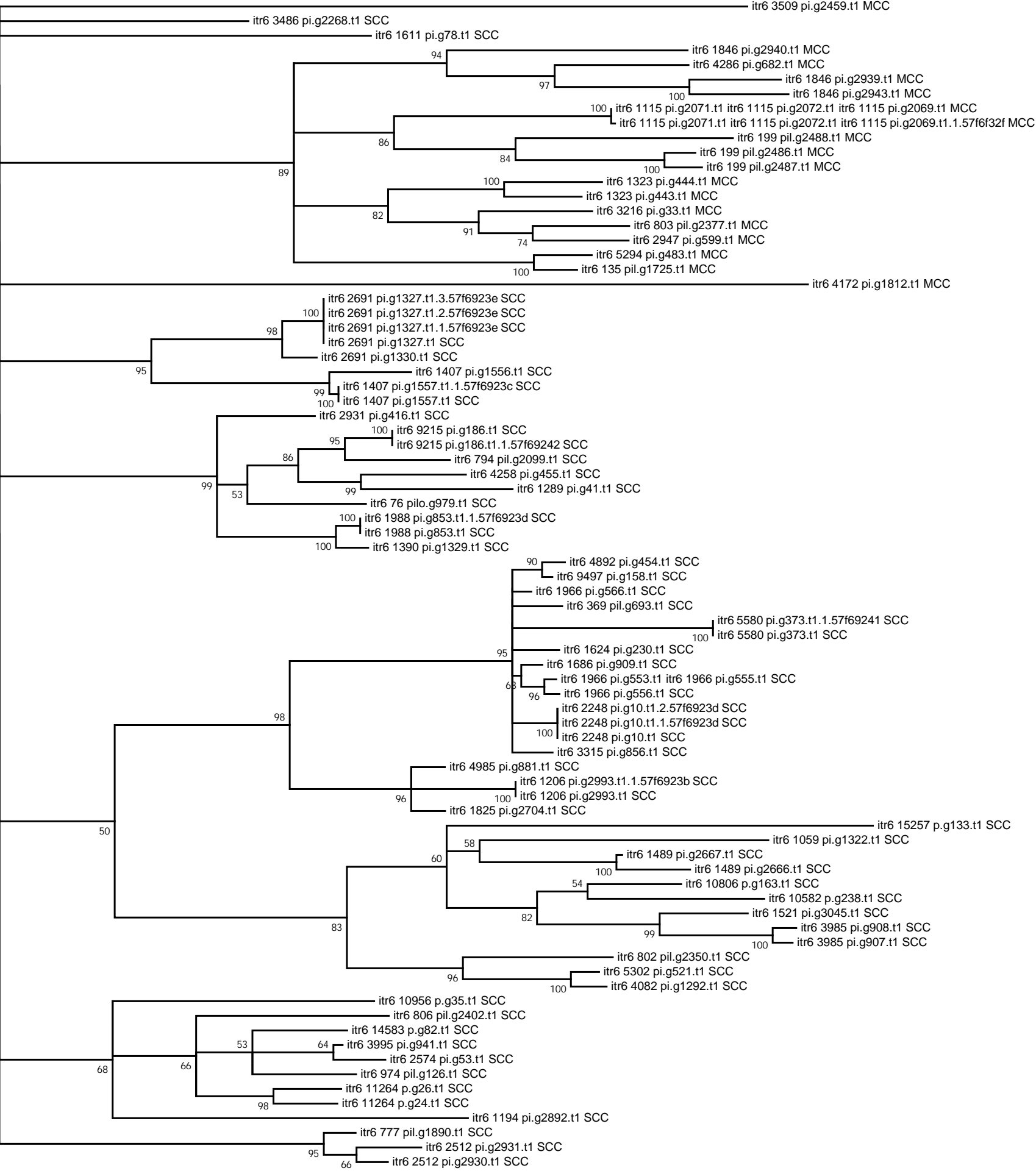

0.2

Supplement: Supplemental material [file gix128_supp.zip › figureS2.pdf]
