# Supplementary material for: A hybrid-hierarchical genome assembly strategy to sequence the invasive golden mussel, Limnoperna fortunei
Source: Gigascience. 2017 Dec 15;7(2):gix128. doi: 10.1093/gigascience/gix128 (PMC5836269; doi:10.1093/gigascience/gix128)
Supplement: Response_to_Reviewer_Comments_Original_Submission.pdf [file gix128_response_to_reviewer_comments_original_submission.pdf]

Author's Response To Reviewer Comments

We thank the reviewers for their attentive read of the manuscript and for suggesting revisions that have increased the overall quality of the data presentation and of the manuscript. Please find bellow each reviewers comment and the answers to them:

Reviewers 1:

Reviewer 1 - Line 49: could the authors provide an extended background to the readers about the arrival of this invasive species in South America?

Response: Yes, the extended background was provided and it's situated in lines 53-55 in the new submission. It is as follows: "... Research suggests that L. fortunei was introduced in South America through ballast water of ships coming from Hong Kong or Korea [2]. It was found for the first time in the estuary of the La Plata River in 1991 [1]."

Reviewer 1 - Line 66: it is maybe better to specify here "freshwater bivalves". Indeed, many other species could be considered as "invasive" in the marine environment, including Mytilus spp.

Response: "Freshwater" was added at line 72.

Reviewer 1 - Line 76: Also, L. fortunei is a mytiloid and other mussel species are known to display an exceptional tolerance to biotic and abiotic contamination, with remarkable capabilities of accumulation and metabolization of toxicants. It is possible that golden mussels share some of these features with marine mussels.

Response: It's true. But we kept the introduction as it was in order to keep it concise and cohesive.

Reviewer 1 - *Lines 96-97: The choice to use three mussels for DNA extraction and sequencing is unclear (unless this is a typo related to the use of 3 mussels for RNA extraction). Why did the authors choose to use this non-standard procedure? Was the genomic DNA extracted from three different specimens pooled in equimolar quantities and used for sequencing? Usually, as heterozygosity might represent a considerable issue, it is desirable to use a single specimen as a reference for genome assembly.

Response: The idea was to sequence only one specimen. But it was not possible due to (i) Illumina DNA library preparation unanticipated problems and (ii) the amount of DNA necessary for PacBio sequencing. The sequencing facility responsible for producing Illumina pair-end and mate pair reads (UNESP) failed to produce the mate pairs in their first attempt, and they asked for more DNA to repeat the library preparation. As we did not have more tissue from the first specimen, we needed to extract more from a second specimen. After that, as we notice the use of only Illumina would not allow us to produce a contiguous high-quality genome, we decided to sequence PacBio. PacBio libraries need a substantial amount of high-molecular-weight-DNA, and to meet this requirements we needed to extract DNA from a third specimen.

To clarify the use of 3 specimens for the construction of the 3 sequencing libraries, a small complement was added to the sentence in line 103-105. It's as follows "... For the genome assembly, a total of 3 individuals were sampled for DNA extraction from gills and to produce the three types of DNA libraries used in this study."

Reviewer 1 - Lines 137-138: Please indicate what the two colors in figure 1 correspond to (I guess to two different k-mer length, but this is not specified neither in the figure itself, nor in its caption. Also, the relative size of the heterozygous peak compared to the homozygous one is particularly remarkable and indicates an extremely high heterozygosity rate, which the authors could estimate and report. This could be linked easily with the subsequent paragraph and the difficulties in assembling such a highly heterozygous genome using short reads only. Please note that these issues have been also encountered by Murgarella and colleagues in the draft assembly of the M. galloprovincialis genome.

Response: We have added the legend on the figures representing the colors. Red represented a the distribution of kmers size 31 and black represented the kmers of size 25. Also, we have estimated the heterozygosity rate of L. fortunei genome to be 2.07%, and we have included this information and some comments between the lines 150-152 It is as follows: "The rate of heterozygosity was estimated to be 2.07% and it was calculated as described by Vij et al. (2016) [18], using as input data the 25-kmer distribution plot for reads from one unique specimen".

And also we did some editings in lines 185-190 . It is as follows "...One main challenge of assembling bivalve genomes lies in the high heterozygosity and amount of repetitive elements these organisms present: (i) the mussels L. fortunei and Modiolus philippinarum and the oyster Crassostrea gigas genomes were estimated to have heterozygosity rates of 2.07%, 2.02 % 1.95% respectively, which is substantially higher than other animal genomes [29], and (ii) repetitive elements correspond to at least 30% of the genomes of all studied bivalves so far (Table 3) [28, 29, 30, 31, 33, 34, 35]. "

Reviewer 1 - *Table 5 and Figure 3 would benefit from the inclusion of a few recently released genomes of other bivalves. Specifically, a much improved version of the Pinctada fucata genome has just been released on Gigascience (the authors could not have access to this resource at the time of writing their manuscript): https://academic.oup.com/gigascience/article/4034775/The-pearl-oyster-Pinctada-fucata-martensii-genome?searchresult=1.
At the same time, the genome of the pectinoid Mizuhopecten yessoensis has also been released (data is available at http://mgb.ouc.edu.cn/pydatabase/download.php).
The genome of the veneroid clam Ruditapes philippinarum is also now available: https://academic.oup.com/gbe/article-lookup/doi/10.1093/gbe/evx096
In this case, while sequence data is not publicly available yet, the authors are willing to share their data upon request.

Response: The 3 new bivalve genomes (P. fucata, M. yessoensis and R. philippinaum) were included in all the comparative analysis of this paper: in Table 3 and Figures 3 and 4. The

previous P. fucata data was replaced, and now comparisons were done with the new assembly presented by Du et al (https://doi.org/10.1093/gigascience/gix059). Table S3 was updated accordingly. And also line 272.

Reviewer 1 - Line 235: "these genomes" should be "these transcriptomes"
Response: It was corrected. Line 234.

Reviewer 1 - Line 251: the authors could add a brief comment about the 58% rate of gene whose expression could be confirmed, stating that this is a reasonable and even expected result, based on the absence of libraries gathered from developmental stages, some adult tissues (i.e. hemocytes) and mussels subjected to different stress (so that inducible gene products might be absent).

Response: The comment was introduced in line 250-255: It is as follows "...Of those, 58% had transcriptional evidence based on RNA Illumina reads (Table S2) re-mapping, rate that was expected since our RNA-Seq libraries were constructed only for 4 tissues of adult golden mussel specimens without any environmental stresses induction (Table 2). Therefore, these libraries lack transcripts for developmental stages, for some other cell types (i.e. hemocytes) and stress-inducible genes. Finally, 67% of the gene models were annotated by homology searches against Uniprot or NCBI NR (Table 6)."

Reviewer 1 - Lines 27-273: "five mussels" should be "five bivalves". Also, this data could be updated using the newly released bivalve genomes I have listed above.

Response: This was corrected and the information, Supl Table S3, and Figure 3 were updated with the new species included in the analysis. Lines 275.

Reviewer 1 - *Line 276: "reconstruct phylogeny" needs to be detailed. What strategy was used (Bayesian, ML, NJ?), what model of molecular evolution, what software? Are the support values displayed in the tree posterior probabilities or bootstrap values?

Response: The methods used were more detailed in lines 277-282. Also, the updated phylogeny was performed including the new data for the P. fucata genome, replacing the old one used, and also including the new data recommended by the review for R. phillapirum and P. yeoensis. It is as follows: " These sequences were used to reconstruct a phylogeny: the single-copy orthologs sequences were concatenated and aligned with CLUSTALW [45] with a resulting alignment of 30755 sites in length (Figure 3B). ProtTest 3.4.2 [46] was used to estimate the best fitting substitution model, which was VT [47]. With this alignment and model we reconstructed the phylogeny using PhyML [48] and 100 bootstrap repetition, the resulting tree is shown on Figure 3B."
.

Reviewer 1 - *Line 301: TIR domains do not necessarily belong to TLRs. More than half of bivalve TIR-DC proteins are indeed intracellular receptors of unknown function (but which are

still likely involved in intracellular immune signaling (see Gerdol et al, DCI 2017). The interpretation of Figure S2 and the discussion contained in lines 303-309 is therefore quite difficult to be evaluated without knowing whether only proteins containing LRRs+TIR or all those containing TIR domains (with and without LRRs) were taken into account. Furthermore, BLAST is not overly useful, by itself, to classify these proteins, as it has been previously demonstrated.
Considering the complexity of this topic and the fact that this goes probably beyond the scopes of this manuscript, the authors could simplify tis section by reporting and expanded complement of TIR-DC proteins and DEATH-domain containing proteins of different nature which, accordingly to the know functions of these domain and existing literature data, are likely to be involved in immune signaling. Overall the expansion of these gene families might suggest an improved resistance to infections. It is however equally curious that other immune-related gene families (e.g. FREPs and C1qDC) seem to be somewhat contracted in figure 4.

Response: Having found LRRs and TIR in the list of over-represented PFAM we looked for TLRs in Blast results, since it was logical to find many of them. However, we were completely aware that not all those Blast hits could represent a genuine TLR, since Blast is heuristically biased towards short High Scoring Pairs (HSP) that could be tagged only to a TIR domain. We, therefore, used SMART (Simple Modular Architecture Research Tool, see http://smart.embl-heidelberg.de/help/smart_about.shtml) to analyze all Blast TLR hits for their modular domain architectures. Only those sequences showing a prototypical TLR architecture were further considered, i.e. N-terminal extracellular leucine-rich repeat (LRR) motifs including either a single or multiple cysteine cluster domain, a C-terminal TIR domain spaced by a single transmembrane-spanning domain (Leulier & Lemaitre, 2008). We know this analysis is not conclusive but TLR expansions in lophotrochozoa were not known until a few years ago when it has been demonstrated in anellida. This finding can contribute to stimulate TLR evolutionary studies. We added some details of the analysis in the body text to explain that those TLR we considered are representative of genuine TLRs.

We have changed a few sentences in the manuscript accordingly. Lines 319-325: It is as follows: "Overall, the expansion of these gene families might suggest an improved resistance to infections. It is, however, equally curious that other immune-related gene families such as Fribinogen_C and C1q seem to be contracted (Supplementary Table S5). This feature may depend on the evolutionary-driven, yet random, fate of the L. fortunei genome and consequence of different specific duplicate genes in other species. Also, other protein families involved in toxin metabolism, especially glutathione based processes and sulfotransferases are clearly contracted (Table S5)."

Reviewer 1 - Line 555: bellow -> below
Response: Thank you, it was corrected. Line 611.

Reviewer 1 - *In Figure 4 legend, it is specified that transposable elements were taken into account. I guess that, depending on the annotation pipeline followed by the different genome sequencing projects these might have been either masked or not, thereby being often excluded

from the final protein set. While the heat map seems to show that TEs are, in general, extremely expanded in Limnoperna, I would be very careful about this claim. This also applies to Table S4. Considering the very high number of gene predictions corresponding to TEs in Limnoperna a particular attention should be also posed into the calculations of under-representation of domains, as these were made based on relative abundance, which would be de facto lowered in Limnoperna if TEs have been masked in the other molluscan genomes.

Response: We agree with this comment, and it was, in fact, a relevant debate among us if we should include or not such retro-domains in the analysis. However, as it seems that such sequences can have a central biological role in shaping some L fortunei genomic features (and maybe physiological ones), we decided to show them even knowing that in other genome studies they might have been kept out or not considered with attention. Indeed, some genomes we used for the new comparison presented in this revised ms, did include TEs in their annotation analysis, e.g. Ruditapes philippinarum, Haliotis discus, Modiolus philippinarum (See Table 5 of the revised ms). The golden mussel genome always outperformed these numbers. However, we tested how considering TE elements in our PFAM analysis might have biased the down-represented features. The reviewer comment has been very appropriate since it can happen and we were not aware of that. Nevertheless, we are confident of the genuinity of our analysis and results. In fact, we made some trials considering a lower total PFAM count value for frequency normalization in other mollusc genomes. When we re-normalized PFAM frequencies at 5% or 10% less counts than before, about 25% and 50% PFAMs are excluded from the original list. Considering that (i) we have estimated about 2500 PFAM countss (nearly 6%); (ii) some other annotations included in the analysis are actually using PFAM associated to TEs; (iii) we used the most conservative false discovery rate procedure, i.e. Bonferroni's; we can conclude that excluding TE from this analysis can be more detrimental than beneficial to the correct functional annotation of the golden mussel genome.

Reviewer 1 - Table S3: "4 other mollusk" -> please correct 4
Response: Table S3 was updated.

Reviewer #2 (Kevin Kocot): Specific comments:
There are too many very short paragraphs. A paragraph should always have at least two sentences. The paragraph spanning lines 58-65 covers two disparate topics and the introduction of the text may need to be reorganized.

Response: we tried to avoid the short paragraph as much as possible. For example, adding a short paragraph to the last line of Table 3, and then deleting it from the manuscript.

Reviewer 2: Why were multiple individuals used?

Response: The idea was to sequence only one specimen. But it was not possible due to (i) Illumina DNA library preparation unanticipated problems and (ii) the amount of DNA necessary for PacBio sequencing. The sequencing facility responsible for producing Illumina pair-end and mate pair reads (UNESP) failed to produce the mate pairs in their first attempt, and they asked for more DNA to repeat the library preparation. As we did not have more tissue from the first specimen, we needed to extract more from a second specimen. After that, as we notice the use of

only Illumina would not allow us to produce a contiguous high-quality genome, we decided to sequence PacBio. PacBio libraries need a substantial amount of high-molecular-weight-DNA, and to meet this requirements we needed to extract DNA from a third specimen.

To clarify the use of 3 specimens for the construction of the 3 sequencing libraries, a small complement was added to the sentence in line 103-105. It's as follows "... For the genome assembly, a total of 3 individuals were sampled for DNA extraction from gills and to produce the three types of DNA libraries used in this study."

Reviewer 2 : The recent Crown of Thorns sea star genome paper (http://www.nature.com/nature/journal/v544/n7649/full/nature22033.html?foxtrotcallback=true) would be an appropriate citation on line 82.
Response: The citation was added. It's now present in line 88.

Reviewer 2: Line 85: Change "U$ " to "USD $"
Response: It was changed in line 91.

Reviewer 2: Lines 166-167: I suggest the authors move this text to the table.
Response: The small paragraph was removed and now it is presented as the last line of Table 3.

Lines 266-273: Despite the name, OrthoMCL does not identify orthologs, it identifies gene families. These are gene family comparisons and not strict orthologs.

Response: Manuscript was edited. Line 268.
