# Supplementary material for: A hybrid-hierarchical genome assembly strategy to sequence the invasive golden mussel, Limnoperna fortunei
Source: Gigascience. 2017 Dec 15;7(2):gix128. doi: 10.1093/gigascience/gix128 (PMC5836269; doi:10.1093/gigascience/gix128)
Supplement: Response_to_Reviewer_Comments_Revision_1.pdf [file gix128_response_to_reviewer_comments_revision_1.pdf]

Author's Response To Reviewer Comments

Reviewer reports:
Reviewer #1: Thank you for providing an updated version of your manuscript and a detailed reply to all my previous concerns. The manuscript now appears to be nearly ready for publication. I still have a few comments, which could require some minor revisions, if the editor will deem them to be necessary.

Overall, the main issue is the use of three different specimens for genome assembly, which is a non-standard procedure which is always preferable to avoid, especially in highly heterozygous genomes. However, given the circumstances, the strategy used is acceptable, since the "mixed" assembly certainly represents a major improvement compared to the assembly performed with Illumina reads only.

With this respect however, the authors could expand a bit their thoughts around line 200, by briefly explaining this issue as a sort of "warning", which could be of help for future sequencing efforts on other bivalve species. In other words, the assembly statistics could have been even better with the preparation of mate-pair and PacBio libraries from the same specimen. Indeed, high heterozygosity might explain why the statistics of the Limnoperna genome are still lower than other genomes of similar size, but with lower heterozygosity rates and, probably, overall complexity (e.g. P. yessoensis), which have been assembled with Illumina reads only. Apart from SNPs and short indels, there is the possibility that particular regions of the genome present large scale rearrangements (i.e. CNVs, large indels and/or inversions), a phenomenon that has been already observed for Ciona savignyii and other species with large effective population size and broad dispersal of gametes by
spawning (no need to report this in the manuscript though). Overall, I think this might partly explain the residual fragmentation of the genome, as the assembly will be complicated by reads originated from highly polymorphic regions across individuals.

Response: We have added a comment about our use of DNA from different specimens and have advised that this is not the ideal way to go (lines: 203-212). However, it's good to note that some genome projects are unable to proceed otherwise. As examples, projects sequencing a rare sample from a threatened species, or from a species for which sample collection and access is difficult. So, it’s good to note that, despite this extra difficulty, the use of hybrid approaches - especially with long reads - can now allow the genome assembly of various species even in these difficult scenarios.

Lines 203-2012 are as follow: “... It’s important to note that assembly statistics can perform better for genomes assembled with reads generated with DNA extracted from one unique individual. This, however, was not possible for L. fortunei’s genome, due to the high amount of high-quality-DNA necessary to produce Illumina mate-pair and PacBio long reads. In this study, the challenge of assembling the high polymorphic regions between haplotypes was enhanced by the difficulties of assembling reads originated from highly polymorphic regions across individuals. However, the golden mussel assembly presented here shows that the use of Illumina contigs, low coverage of PacBio long reads, transcriptome and Illumina re-mapping for final correction (Figure 2) represents an option for cost-efficient assembly of highly heterozygous

genomes of nonmodel species such as bivalves. "

Reviewer 1: Thanks for including k-mer size in figure 1. However, for highly heterozygous genomes the use of shorter k-mers (17-20) is often appropriate for a better estimate of heterozygosity rates (the formula assumes the k-mers falling in the heterozygous peak differ from those of the homozygous peak just by 1 nucleotide, but this assumption might not be correct when long k-mers are used in highly heterozygous genomes). Please try to calculate the rate also with a shorter k-mer size (and plot it in figure 1, if necessary) and check whether the calculated heterozygosity rate changes significantly (it is possible that you are slightly underestimating it with the current k-mer size).

Response: We have updated the heterozygosity rate estimated with kmer size of 17 in lines 153 and 190 of the new manuscript.

Reviewer 1: Lines 277-282: please check the log file of your ProtTest analysis. The selection of the VT model (without a +G, or +F parameter) is somewhat odd. It is possible that your machine ran out of memory during the computation of the most complex models due to the large size of the input alignment, so that the LogL values could be computed just for the most simple models (such as VT). In any case the tree topology is exactly that one might expect, so the possible use of a different model will only have subtle effects.

Response: ProtTest selected the VT+G+I+F model, and this was what we used in our phylogeny (line 286).

Reviewer 1: Same % values are missing in table 5 for the newly added genomes. Also, check the comas to indicate thousands in all numbers.

Response: the table was re-checked and corrected for %s and commas. Thank you.
