# Supplementary material for: A hybrid-hierarchical genome assembly strategy to sequence the invasive golden mussel, Limnoperna fortunei
Source: Gigascience. 2017 Dec 15;7(2):gix128. doi: 10.1093/gigascience/gix128 (PMC5836269; doi:10.1093/gigascience/gix128)
Supplement: Reviewer_1_Report_(Original_Submission) -- Marco Gerdol — 31 Jul 2017 Reviewed [file gix128_reviewer_1_report_(original_submission).pdf]

Reviewer Report

Title: A hybrid-hierarchical genome assembly strategy to sequence the invasive golden mussel Limnoperna fortunei

Version: Original Submission **Date:** 7/31/2017

Reviewer name: Marco Gerdol

Reviewer Comments to Author:

The manuscript submitted for consideration by Uliano-Silva and colleagues report the sequencing, assembly and annotation of the genome of Limnoperna fortunei, an invasive mussel species which is the cause of serious concern in South America. Since the authors chose to submit this manuscript as a "data note", in this review report I will mainly evaluate the technical aspects of the work and the correctness and completeness of the biological context provided by the authors. However, since the authors have also partially discussed some biological implications of their main findings, I will also suggest potential improvements whenever needed.Based on the guidelines for assessing the merit of "data note" manuscripts submitted for consideration to GigaScience:How the data meets the FAIR (Findable, Accessible, Interoperable and Reusable) principlesThe data fully meet the abovementioned criteria.Rarity or unusual nature of data type.Genomic resources for bivalve mollusks are still very scarce and any new addition is welcome, especially considering the peculiar genetic features of these species which have long complicated studies in this field.Novel technology or methodology used to create dataset.The authors employed an appropriate methodology, combining Illumina and PacBio sequencing. Considering the high heterozygosity of this genome, this strategy was a well-planned choice.Need for immediate public health issues.Not applicable.Reuse potential.This data has a high reuse potential, both for targeted studies for the management of this invasive species and for comparative genomics studies in bivalves.Despite the usefulness of this resource and the valid methodology used by the authors, in my opinion a number of issues need to be addressed before this manuscript can be accepted for publication on GigaScience.Please find my detailed evaluation below, with major issues marked by an asterisk.Line 49: could the authors provide an extended background to the readers about the arrival of this invasive species in South America?Line 66: it is maybe better to specify here "freshwater bivalves". Indeed, many other species could be considered as "invasive" in the marine environment, including Mytilus spp.Line 76: Also, L. fortunei is a mytiloid and other mussel species are known to display an exceptional tolerance to biotic and abiotic contamination, with remarkable capabilities of accumulation and metabolization of toxicants. It is possible that golden mussels share some of these features with marine mussels.*Lines 96-97: The choice to use three mussels for DNA extraction and sequencing is unclear (unless this is a typo related to the use of 3 mussels for RNA extraction). Why did the authors choose to use this non-standard procedure? Was the genomic DNA extracted from three different specimens pooled in equimolar quantities and used for sequencing? Usually, as heterozygosity might represent a considerable issue, it is desirable to use a single specimen as a reference for genome assembly.Lines 137-138: Please indicate what the two colors in figure 1 correspond to (I guess to two different k-mer length, but this is not specified neither in the figure itself, nor in its caption. Also, the relative size of the

heterozygous peak compared to the homozygous one is particularly remarkable and indicates an extremely high heterozygosity rate, which the authors could estimate and report. This could be linked easily with the subsequent paragraph and the difficulties in assembling such a highly heterozygous genome using short reads only. Please note that these issues have been also encountered by Murgarella and colleagues in the draft assembly of the M. galloprovincialis genome.*Table 5 and Figure 3 would benefit from the inclusion of a few recently released genomes of other bivalves. Specifically, a much improved version of the Pinctada fucata genome has just been released on Gigascience (the authors could not have access to this resource at the time of writing their manuscript): https://academic.oup.com/gigascience/article/4034775/The-pearl-oyster-Pinctada-fucata-martensii-genome?searchresult=1.At the same time, the genome of the pectinoid Mizuhopecten yessoensis has also been released (data is available at http://mgb.ouc.edu.cn/pydatabase/download.php).The genome of the veneroid clam Ruditapes philippinarum is also now available: https://academic.oup.com/gbe/article-lookup/doi/10.1093/gbe/evx096In this case, while sequence data is not publicly available yet, the authors are willing to share their data upon request.Line 172: slightest -> slightlyLine 235: "these genomes" should be "these transcriptomes"Line 251: the authors could add a brief comment about the 58% rate of gene whose expression could be confirmed, stating that this is a reasonable and even expected result, based on the absence of libraries gathered from developmental stages, some adult tissues (i.e. hemocytes) and mussels subjected to different stress (so that inducible gene products might be absent).Lines 272-273: "five mussels" should be "five bivalves". Also, this data could be updated using the newly released bivalve genomes I have listed above.*Line 276: "reconstruct phylogeny" needs to be detailed. What strategy was used (Bayesian, ML, NJ?), what model of molecular evolution, what software? Are the support values displayed in the tree posterior probabilities or bootstrap values?*Line 301: TIR domains do not necessarily belong to TLRs. More than half of bivalve TIR-DC proteins are indeed intracellular receptors of unknown function (but which are still likely involved in intracellular immune signaling (see Gerdol et al, DCI 2017). The interpretation of Figure S2 and the discussion contained in lines 303-309 is therefore quite difficult to be evaluated without knowing whether only proteins containing LRRs+TIR or all those containing TIR domains (with and without LRRs) were taken into account. Furthermore, BLAST is not overly useful, by itself, to classify these proteins, as it has been previously demonstrated.Considering the complexity of this topic and the fact that this goes probably beyond the scopes of this manuscript, the authors could simplify tis section by reporting and expanded complement of TIR-DC proteins and DEATH-domain containing proteins of different nature which, accordingly to the know functions of these domain and existing literature data, are likely to be involved in immune signaling. Overall the expansion of these gene families might suggest an improved resistance to infections. It is however equally curious that other immune-related gene families (e.g. FREPs and C1qDC) seem to be somewhat contracted in figure 4.Line 555: bellow -> below*In Figure 4 legend, it is specified that transposable elements were taken into account. I guess that, depending on the annotation pipeline followed by the different genome sequencing projects these might have been either masked or not, thereby being often excluded from the final protein set. While the heat map seems to show that TEs are, in general, extremely expanded in Limnoperna, I would be very careful about this claim. This also applies to Table S4. Considering the very high number of gene predictions corresponding to TEs in Limnoperna a particular attention should be also posed into the calculations of under-representation of domains, as these were made based on relative abundance,

which would be de facto lowered in Limnoperna if TEs have been masked in the other molluscan genomes.Table S3: "4 other mollusk" -> please correct 4

Level of Interest

Please indicate how interesting you found the manuscript: An article of importance in its field

Quality of Written English

Please indicate the quality of language in the manuscript: Acceptable

Declaration of Competing Interests

Please complete a declaration of competing interests, considering the following questions:

- Have you in the past five years received reimbursements, fees, funding, or salary from an organisation that may in any way gain or lose financially from the publication of this manuscript, either now or in the future?
- Do you hold any stocks or shares in an organisation that may in any way gain or lose financially from the publication of this manuscript, either now or in the future?
- Do you hold or are you currently applying for any patents relating to the content of the manuscript?
- Have you received reimbursements, fees, funding, or salary from an organization that holds or has applied for patents relating to the content of the manuscript?
- Do you have any other financial competing interests?
- Do you have any non-financial competing interests in relation to this paper?

If you can answer no to all of the above, write 'I declare that I have no competing interests' below. If your reply is yes to any, please give details below.

I declare that I have no competing interests

I agree to the open peer review policy of the journal. I understand that my name will be included on my report to the authors and, if the manuscript is accepted for publication, my named report including any attachments I upload will be posted on the website along with the authors' responses. I agree for my report to be made available under an Open Access Creative Commons CC-BY license (http://creativecommons.org/licenses/by/4.0/). I understand that any comments which I do not wish to be included in my named report can be included as confidential comments to the editors, which will not be published.

I agree to the open peer review policy of the journal
