# Supplementary material for: A hybrid-hierarchical genome assembly strategy to sequence the invasive golden mussel, Limnoperna fortunei
Source: Gigascience. 2017 Dec 15;7(2):gix128. doi: 10.1093/gigascience/gix128 (PMC5836269; doi:10.1093/gigascience/gix128)
Supplement: Reviewer_1_Report_(Revision_1) -- Marco Gerdol — 08 Nov 2017 Reviewed [file gix128_reviewer_1_report_(revision_1).pdf]

Reviewer Report

Title: A hybrid-hierarchical genome assembly strategy to sequence the invasive golden mussel Limnoperna fortunei

Version: Revision 1 **Date:** 11/8/2017

Reviewer name: Marco Gerdol

Reviewer Comments to Author:

Thank you for providing an updated version of your manuscript and a detailed reply to all my previous concerns. The manuscript now appears to be nearly ready for publication. I still have a few comments, which could require some minor revisions, if the editor will deem them to be necessary.Overall, the main issue is the use of three different specimens for genome assembly, which is a non-standard procedure which is always preferable to avoid, especially in highly heterozygous genomes. However, given the circumstances, the strategy used is acceptable, since the "mixed" assembly certainly represents a major improvement compared to the assembly performed with Illumina reads only.With this respect however, the authors could expand a bit their thoughts around line 200, by briefly explaining this issue as a sort of "warning", which could be of help for future sequencing efforts on other bivalve species. In other words, the assembly statistics could have been even better with the preparation of mate-pair and PacBio libraries from the same specimen. Indeed, high heterozygosity might explain why the statistics of the Limnoperna genome are still lower than other genomes of similar size, but with lower heterozygosity rates and, probably, overall complexity (e.g. P. yessoensis), which have been assembled with Illumina reads only. Apart from SNPs and short indels, there is the possibility that particular regions of the genome present large scale rearrangements (i.e. CNVs, large indels and/or inversions), a phenomenon that has been already observed for Ciona savignyii and other species with large effective population size and broad dispersal of gametes by spawning (no need to report this in the manuscript though). Overall, I think this might partly explain the residual fragmentation of the genome, as the assembly will be complicated by reads originated from highly polymorphic regions across individuals.Thanks for including k-mer size in figure 1. However, for highly heterozygous genomes the use of shorter k-mers (17-20) is often appropriate for a better estimate of heterozygosity rates (the formula assumes the k-mers falling in the heterozygous peak differ from those of the homozygous peak just by 1 nucleotide, but this assumption might not be correct when long k-mers are used in highly heterozygous genomes). Please try to calculate the rate also with a shorter k-mer size (and plot it in figure 1, if necessary) and check whether the calculated heterozygosity rate changes significantly (it is possible that you are slightly underestimating it with the current k-mer size).Lines 277-282: please check the log file of your ProtTest analysis. The selection of the VT model (without a +G, or +F parameter) is somewhat odd. It is possible that your machine ran out of memory during the computation of the most complex models due to the large size of the input alignment, so that the LogL values could be computed just for the most simple models (such as VT). In any case the tree topology is exactly that one might expect, so the possible use of a different model will only have subtle effects.The procedure used for the detection of domain expansion is now very reasonable. As a further comment that I forgot to mention in

my first report, it is also possible that some mobile elements present in multiple copies in various genomes have been collapsed in a single or a very few copies in Illumina-based assemblies, whereas the Limnoperna genome, with the use of long reads, correctly represents most of them as separate genes, inflating a bit the expansion scores. No need to change anything from this side in the manuscript.Same % values are missing in table 5 for the newly added genomes. Also, check the comas to indicate thousands in all numbers.

Level of Interest

Please indicate how interesting you found the manuscript: An article of importance in its field

Quality of Written English

Please indicate the quality of language in the manuscript: Acceptable

Declaration of Competing Interests

Please complete a declaration of competing interests, considering the following questions:

- Have you in the past five years received reimbursements, fees, funding, or salary from an organisation that may in any way gain or lose financially from the publication of this manuscript, either now or in the future?
- Do you hold any stocks or shares in an organisation that may in any way gain or lose financially from the publication of this manuscript, either now or in the future?
- Do you hold or are you currently applying for any patents relating to the content of the manuscript?
- Have you received reimbursements, fees, funding, or salary from an organization that holds or has applied for patents relating to the content of the manuscript?
- Do you have any other financial competing interests?
- Do you have any non-financial competing interests in relation to this paper?

If you can answer no to all of the above, write 'I declare that I have no competing interests' below. If your reply is yes to any, please give details below.

I declare that I have no competing interests

I agree to the open peer review policy of the journal. I understand that my name will be included on my report to the authors and, if the manuscript is accepted for publication, my named report including any attachments I upload will be posted on the website along with the authors' responses. I agree for my report to be made available under an Open Access Creative Commons CC-BY license (http://creativecommons.org/licenses/by/4.0/). I understand that any comments which I do not wish to

be included in my named report can be included as confidential comments to the editors, which will not be published.

I agree to the open peer review policy of the journal
