# Supplementary material for: A hybrid-hierarchical genome assembly strategy to sequence the invasive golden mussel, Limnoperna fortunei
Source: Gigascience. 2017 Dec 15;7(2):gix128. doi: 10.1093/gigascience/gix128 (PMC5836269; doi:10.1093/gigascience/gix128)
Supplement: Reviewer_2_Report_(Original_Submission) -- Kevin Kocot — 23 Aug 2017 Reviewed [file gix128_reviewer_2_report_(original_submission).pdf]

Reviewer Report

Title: A hybrid-hierarchical genome assembly strategy to sequence the invasive golden mussel Limnoperna fortunei

Version: Original Submission **Date:** 8/23/2017

Reviewer name: Kevin Kocot

Reviewer Comments to Author:

This is a high quality Data Note describing a new genomic resource for the invasive golden mussel (Limnoperna fortunei). The assembly and annotation follow industry standard protocols and are a good example of how to combine Illumina and PacBio data to produce a high quality draft genome for a non-model organism, of interest to workers in broad fields. The manuscript is well written (although I have some suggestions for improvement; see below).Specific comments:There are too many very short paragraphs. A paragraph should always have at least two sentences. The paragraph spanning lines 58-65 covers two disparate topics and the introduction of the text may need to be reorganized.Why were multiple individuals used?The recent Crown of Thorns sea star genome paper (http://www.nature.com/nature/journal/v544/n7649/full/nature22033.html?foxtrotcallback=true) would be an appropriate citation on line 82.Line 85: Change "U$ " to "USD $"Lines 166-167: I suggest the authors move this text to the table.Lines 266-273: Despite the name, OrthoMCL does not identify orthologs, it identifies gene families. These are gene family comparisons and not strict orthologs.

Level of Interest

Please indicate how interesting you found the manuscript: An article of importance in its field

Quality of Written English

Please indicate the quality of language in the manuscript: Acceptable

Declaration of Competing Interests

Please complete a declaration of competing interests, considering the following questions:

- Have you in the past five years received reimbursements, fees, funding, or salary from an organisation that may in any way gain or lose financially from the publication of this manuscript, either now or in the future?
- Do you hold any stocks or shares in an organisation that may in any way gain or lose financially from the publication of this manuscript, either now or in the future?
- Do you hold or are you currently applying for any patents relating to the content of the manuscript?

- Have you received reimbursements, fees, funding, or salary from an organization that holds or has applied for patents relating to the content of the manuscript?
- Do you have any other financial competing interests?
- Do you have any non-financial competing interests in relation to this paper?

If you can answer no to all of the above, write 'I declare that I have no competing interests' below. If your reply is yes to any, please give details below.

I declare that I have no competing interests

I agree to the open peer review policy of the journal. I understand that my name will be included on my report to the authors and, if the manuscript is accepted for publication, my named report including any attachments I upload will be posted on the website along with the authors' responses. I agree for my report to be made available under an Open Access Creative Commons CC-BY license (http://creativecommons.org/licenses/by/4.0/). I understand that any comments which I do not wish to be included in my named report can be included as confidential comments to the editors, which will not be published.

I agree to the open peer review policy of the journal
